# The risk elicitation puzzle revisited: Across-methods (in)consistency?

**DOI:** 10.1007/s10683-020-09674-8

**Published:** 2020-09-12

**Authors:** Felix Holzmeister, Matthias Stefan

**Affiliations:** 1 Department of Economics, University of Innsbruck, Innsbruck, Austria; 2 Department of Banking and Finance, University of Innsbruck, Innsbruck, Austria

**Keywords:** Risk preference elicitation, Inconsistent behavior, Risk attitudes, C91, D81

## Abstract

With the rise of experimental research in the social sciences, numerous methods to elicit and classify people’s risk attitudes in the laboratory have evolved. However, evidence suggests that attitudes towards risk may vary considerably when measured with different methods. Based on a within-subject experimental design using four widespread risk preference elicitation tasks, we find that the different methods indeed give rise to considerably varying estimates of individual and aggregate level risk preferences. Conducting simulation exercises to obtain benchmarks for subjects’ behavior, we find that the observed heterogeneity in risk preference estimates across methods is qualitatively similar to the heterogeneity arising from independent random draws from the choice distributions observed in the experiment. Our study, however, provides evidence that subjects are surprisingly well aware of the variation in the riskiness of their choices. We argue that this calls into question the common interpretation of variation in revealed risk preferences as being inconsistent.

**Electronic supplementary material:**

The online version of this article (10.1007/s10683-020-09674-8) contains supplementary material, which is available to authorized users.

## Introduction

Risk is an integral part of many economic decisions and, thus, has been considered a key building block of economic theory (Arrow 1965). As a consequence, the question how to properly elicit and classify individuals’ risk preferences is of vital importance in academic research. In experimental economics and psychology, irrespective of differences in their approaches, incentivized risk preference elicitation tasks have evolved as widely accepted tools to measure and assess individual-level attitudes towards risk. While economists and psychologists have developed a variety of competing methodologies, a consensus on which of the elicitation procedures gives rise to the most accurate estimates of individual-level risk preferences has not been reached yet (Charness et al. 2013). Facing this pluralism of methods, pragmatism prevails among researchers when choosing among various competing risk preference elicitation tasks. The implicit assumption behind this common practice is the procedural invariance axiom, which states that normatively equivalent elicitation methods give rise to the same preference ordering (Tversky et al. 1988). Accordingly, the experimenter’s choice of which method to use should not systematically affect participants’ revealed risk preferences. However, experimental evidence, reviewed in detail in Sect. 2, suggests that participants’ attitudes towards risk may vary considerably when measured with different elicitation methods—a finding recently referred to as the “risk elicitation puzzle” (Pedroni et al. 2017).

What is particularly challenging about the risk elicitation puzzle is not the heterogeneity in risk preferences across different methods per se, but rather the question how to properly interpret the observed variation in risk attitudes. In particular, how can we assess whether choices that can be described by varying risk preferences are indeed the result of unstable preferences, or, whether different elicitation methods rather stimulate distinct preference relations? While the former interpretation challenges the assumption of stable risk preferences, the latter challenges the procedural invariance axiom; and indeed, calling procedural invariance into question dates back to early systematic examinations of preference reversals (see, e.g., Tversky et al. 1988; Tversky and Thaler 1990). A third option is to adhere to both assumptions, i.e., preference stability and procedural invariance, but rather interpret subjects’ behavior as inconsistent—a term abundantly used in the literature with various meanings. However, it is not immediately obvious what the term *inconsistent* should refer to in terms of choice behavior. As argued by Sen (1993), “the basic difficulty arises from the implicit presumption underlying that approach that acts of choices are, on their own, like statements which can contradict, or be consistent with, each other.” Thus, to assess the consistency of behavior, eventually, one needs to invoke a theory upon which choices can be interpreted as contradictory (Sugden 1991). This essential insight illustrates that one can only assess the consistency of choices across different methods on the basis of some underlying theoretical framework. Part of this framework are the premises of preference stability and procedural invariance, which allow for evaluating participants’ behavior as inconsistent under the assumption that different methods elicit the same stable preference relation. If either of the two premises is waived, however, classifying heterogeneity in revealed risk preferences as inconsistent becomes questionable. While we can conceptually disentangle preference stability from procedural invariance, it is important to emphasize that the validity of either of the two premises cannot be tested in isolation. Any test of either concept involves the assumption of the other: Examining the stability of preferences requires the usage of different risk preference elicitation methods to compare the elicited preferences, which (implicitly) assumes procedural invariance—and *vice versa*.^1^

To get a better understanding of variability of revealed preferences across methods, in this paper we take into account participants’ subjective point of view: In addition to incentivized risk preference elicitation tasks, our experimental protocol comprises survey items, which allow for examining participants’ subjective accounts of the different methods—in particular, their awareness of the risk they are willing to take in the different tasks. We use a within-subject design comprising four widely used risk preference elicitation methods: (1) the “bomb” risk elicitation task (Crosetto and Filippin 2013), (2) the certainty equivalent method (Cohen et al. 1987; Dohmen et al. 2010; Abdellaoui et al. 2011), (3) a multiple choice list between pairs of lotteries (Holt and Laury 2002, 2005), and (4) a single choice list (Binswanger 1980, 1981; Eckel and Grossman 2002, 2008). While previous studies typically assess the magnitude of across-methods variation based on correlations between risky choices in different tasks, we employ an individual-level measure of preference stability relying on the comparison of implied crra parameter intervals. For our sample, we observe that subjects’ revealed preferences are stable in less than 50% of pairwise comparisons of methods. Conducting simulation exercises to obtain benchmarks for participants’ behavior, we find that the observed heterogeneity of revealed risk preference across methods is qualitatively similar to the heterogeneity arising from independent random draws from choices in the experimental tasks. While this finding is indicative of substantial across-methods variation in risk-taking behavior, our main result is that subjects’ assessments of the riskiness of their choices is significantly related to the risk preference estimates across the different tasks. Thus, subjects seem to be well aware of their choices across methods. In the light of these results, we argue that the observed variation in revealed preferences cannot be straightforwardly interpreted as being inconsistent.

## Related literature

The question whether different experimental procedures to measure individual-level risk attitudes give rise to the same revealed preferences dates back more than 50 years.^2^ Slovic (1964), to the best of our knowledge, was first to challenge the standard assumption of procedural invariance by concluding that “the domain of risk taking behavior may not be as conceptually unitary as many psychologists would like to believe.” An early study by Slovic (1972a) comparing attitudes towards risk using two different procedures corroborates the skepticism about method invariance by emphasizing low levels of inter-measure correlation. Slovic (1972a, b) argues that different procedures trigger different processing of information about probabilities and payoffs, and that situation specificity is a crucial dimension of risk-taking behavior.

Almost three decades later, the question whether risk preferences are properly modelled as a generally stable personality trait has been revisited. Using a first price auction and the Becker-DeGroot-Marschak procedure (bdm; Becker et al. 1964), Isaac and James (2000) find that the rank-order of revealed preferences across individuals is not preserved across the two institutions. Berg et al. (2005) substantiate these results in a non-parametric framework, comparing revealed risk preferences in a bdm mechanism, an English clock auction, and a first price auction. In a similar manner, several more recent studies investigate across-methods heterogeneity in revealed risk preferences utilizing multiple price list formats. Anderson and Mellor (2009) show that subjects do not reveal stable risk preferences across an incentivized price list (hl; Holt and Laury 2002) and an unincentivized survey on hypothetical gambles. Bruner (2009) reports pronounced variability in risky choices in two price lists with the same expected payoffs, only altering whether lotteries vary in payoff or probability. Hey et al. (2009) examine the variability of revealed preferences across four different elicitation methods and conclude that the differences in the methods’ noisiness and bias might account for observed variation. Dave et al. (2010) and Reynaud and Couture (2012) compare risk preferences estimated with the hl method and the single choice list procedure introduced by Eckel and Grossman (2002). Both studies report substantial differences in estimated risk attitudes. While Dave et al. (2010) suggest that inter-subject differences in risk preference estimates can partly be attributed to a lack of numeracy, Reynaud and Couture (2012) argue that the variation in risk preferences across methods relates to non-expected utility preferences (Starmer 2000) and context-dependent preferences (Weber et al. 2002).

Relating to this discussion, Dohmen et al. (2011) find that participants’ willingness to take risk varies with context, but is largely correlated. They suggest that the elicited measures of risk preferences contain a context-specific component, but also a common trait that underlies the choices in different contexts. In a similar vein, Lévy-Garboua et al. (2012) provide evidence that the degree of heterogeneity in risky choices varies for different frames of the same lottery choice experiment (see also Meraner et al. 2018). Deck et al. (2013) do not find evidence that domain specificity explains the observed variation in revealed risk preferences across four elicitation methods and additional survey questions. Relating to the discussion of risk preferences as a stable trait, Frey et al. (2017) report experimental evidence that a general factor of risk preference explains a substantial part of the variation in questionnaires, but less so in experimental methods (see also Mata et al. 2018).

Alternative explanations of the observed variability in risk preferences across tasks are provided in a between-subject analysis by Crosetto and Filippin (2015). Even accounting for task-specific measurement errors, they report substantial variation in risk preference estimates across four elicitation methods and discuss potential explanations based on the availability of a safe option and the difference between a single- and a multiple-choice environment. Pedroni et al. (2017) find substantial variation in risky choices across six risk elicitation mechanisms even when controlling for measurement errors and subjects’ numeracy. Furthermore, they do not find support for the assumption that different subjects consistently decide according to Expected Utility or Prospect Theory across tasks. In a recent study with six elicitation methods, Friedman et al. (2018) find that an expected utility framework decently explains subject behavior in revealing risk preferences except for across-methods variation. The authors further report that part of the observed heterogeneity can be explained by characteristics of the elicitation methods, such as spatial representation or whether prices or probabilities are varied. Similarly, using two risk elicitation methods by Wakker and Deneffe (1996) and Tanaka et al. (2010), Bauermeister et al. (2018) not only report heterogeneity in revealed preferences, but also in probability weightings.

Overall, the previous literature on the across-methods variability of revealed preferences tends to agree that the heterogeneity in risk preferences is substantial. While the correlations between risky choices in pairwise comparisons of methods, on average, tend to be positive, correlation coefficients span a wide range: The approximately 90 pairwise correlation coefficients reported in the studies discussed above vary from − 0.33 (Isaac and James 2000) to 0.86 (Friedman et al. 2018), leaving the reader with rather inconclusive insights about the actual extent of the across-methods variability of risk preferences. Since it is not clear how to interpret the empirically observed variation in elicited risk attitudes, the primary goal of our study is not to add to the pile of evidence of seemingly inconsistent behavior, but rather to contribute to the understanding of the observed across-method variation in risk preferences. Our main contribution to the literature is to argue that participants in our experiment are well aware of the riskiness associated with their choices and, thus, that their behavior should not be readily interpreted as inconsistent.

## Experimental design

We conducted ten experimental sessions with a total of 198 participants (55% female; age: $m = 22.9$ years, $sd = 2.5$) in the *Innsbruck EconLab*. The experiment was computerized using *oTree* (Chen et al. 2016), utilizing the ready-made applications for risk preference elicitation methods by Holzmeister and Pfurtscheller (2016) and Holzmeister (2017). Participants—bachelor and master students from various fields of study—were recruited using hroot (Bock et al. 2014). Upon arrival in the laboratory, participants were seated randomly and asked to start the experiment after having carefully read the instructions on screen. Experimental sessions were conducted in German, took approximately 40 min, and were all administered by the same experimenters. Participants received an average payment of €21.35 including a show-up fee of €4.00 (*sd* = €6.25, *min* = €8.00, *max* = €38.50).

We used a within-subject design to measure individual-level risk preferences in four different risk elicitation methods, all of which are commonly applied in social science experiments: (1) the “bomb” risk elicitation task (bret), (2) the certainty equivalent method (cem), (3) a multiple choice list between pairs of lotteries (mpl), and (4) a single choice list (scl). Since numerous methods have been introduced to measure risk preferences in the lab, our selection necessarily involves a moment of arbitrariness. However, the four risk preference elicitation tasks included in our study continue to be among the most popular and most widely used ones. Thus, we deem our choice a good starting point for our analysis.

The parametrization of each task has been mapped to the lottery payoffs and probabilities proposed in the original articles but were scaled in such a way that the expected payoffs of a risk neutral decision maker are similar across tasks (approximately €12.00). The instructions for each of the elicitation methods were displayed just before participants were asked to make their choice(s) in the particular decision problem. Translated instructions and screenshots of the entire experiment are provided in Appendix 7 in Electronic Supplementary Material.

To avoid order and learning effects across tasks (see, e.g., Carlsson et al. 2012), each participant faced a random sequence of the four risk preference elicitation methods.^3^ To avoid portfolio-building and cross-task contamination effects (see, e.g., Cubitt et al. 1998; Harrison and Ruström 2008), a random lottery incentive system was implemented, i.e., only one of the four tasks was randomly chosen for a subject’s final payment (Azrieli et al. 2018).^4^ A persistent phenomenon in choice list elicitation procedures is the observation of multiple switching behavior (see, e.g., Bruner 2011), violating monotonicity and transitivity of revealed preferences and, thus, the paradigm of utility maximization. As our intent is to examine (in)consistency *between* rather than within tasks, we enforced a single switching point in the two multiple price list tasks (cem and mpl) as proposed by Andersen et al. (2006) and utilized by Jacobson and Petrie (2009) and Tanaka et al. (2010) among others.^5^

### Elicitation methods

In the following, (*x*, *p*; *y*) denotes a two-outcome lottery that assigns probability *p* to outcome *x* and probability $1-p$ to outcome *y*. Subscripts *h* and *l* refer to “high” and “low” lottery outcomes, respectively.

*The “bomb” risk elicitation task (bret)* The bret is a visual risk preference elicitation method requiring subjects to decide on how many boxes to collect out of a matrix containing *n* boxes. Each box collected yields a payoff $\gamma$; but in one of the boxes a “bomb” is hidden, destroying all prospective earnings. Thus, potential earnings increase linearly, but are zero if the bomb is contained in one of the collected boxes. By this means, the bret elicits (within-method consistent) decisions in $n+1$ lotteries ($\gamma k$, ${(n-k)}/{n}$; 0), and measures individual-level risk attitudes by a single parameter $k \in \lbrace 0, 1, \ldots , n \rbrace$, the number of boxes collected. As in Crosetto and Filippin (2013), boxes were collected dynamically and randomly with a time interval of one second for each box once the “Start” button was hit until the “Stop” button was hit.^6^ The location of the bomb is only revealed at the end of the task. In our experiment, we set *n* to 100 and $\gamma$ to €0.50, implying an expected payoff of €12.50 for a risk neutral decision maker.

*Certainty equivalent method (cem)* The cem elicits the point of indifference between a fixed risky lottery $L^A$ = ($a_h$, *p*; $a_l$) with $a_h > a_l$ and *n* varying degenerate lotteries, i.e., sure payoffs $L^B_i$ = ($b_i$, 1), with $a_h \ge b_i \ge a_l$ for all $i = 1,2,\ldots ,n$. We implement the parametrization used by Abdellaoui et al. (2011) with $n = 9$ binary choices, scaled by a factor of 0.5, i.e., $a_h =$ €15.00, $a_l =$ €5.00, and $b_i = \lbrace$ €5.00, €6.25, $\ldots$, €15.00 $\rbrace$. A risk neutral subject expects to earn €11.39.

*Multiple price list* (mpl) The mpl is characterized by a set of ten binary choices between lotteries with fixed payoffs but varying probabilities of high and low outcomes for each choice. That is, subjects face a menu of *n* binary choices between lottery $L^A_i$ = ($a_h$, $p_i$; $a_l$) and lottery $L^B_i$ = ($b_h$, $p_i$; $b_l$) for $i = 1,2,\ldots ,n$, where $b_h> a_h> a_l > b_l$. We use the parametrization with $n = 10$ lotteries as proposed by Holt and Laury (2002) but scaled the payoffs by a factor of 5, i.e., $a_h =$ €19.25, $a_l =$ €0.50, $b_h =$ €10.00, and $b_l =$ €8.00 with $p_i = \lbrace 0.10, 0.20, \ldots , 1.00 \rbrace$. A risk neutral individual expects a payoff of €12.14.

*Single choice list* (scl) The scl offers subjects a menu of different lotteries, asking them to choose the one they prefer to be played. The menu consists of six lotteries which are similar to the implementation proposed by Eckel and Grossman (2002, (2008): $L_1$ = (€9.00, 0.50; €9.00), $L_2$ = (€7.50, 0.50; €12.00), $L_3$ = (€6.00, 0.50; €15.00), $L_4$ = (€4.50, 0.50; €18.00), $L_5$ = (€3.00, 0.50; €21.00), and $L_6$ = (€0.00, 0.50; €24.00). Note that lotteries $L_5$ and $L_6$ have the same expected payoff but differ in their standard deviation. That is, choosing $L_5$ implies that the decision maker is either (weakly) risk averse or risk-neutral; choosing $L_6$ reveals risk neutrality or risk seeking preferences. Hence, a risk neutral decision maker chooses either lottery $L_5$ or lottery $L_6$, implying an expected payoff of €12.00.

### Questionnaires

To relate the observed behavior in the four risk preference elicitation methods to subjects’ perception of the tasks’ characteristics as well as their comprehension and numeracy, the experimental protocol comprised several additional questionnaires. Details on the questionnaires and subjects’ responses are provided in “Appendices 1–3” in Electronic Supplementary Material. Our approach of combining experimental with questionnaire data is somewhat exploratory in nature. However, given the vast number of puzzling findings on the (in)stability of risk preferences in the literature and the lack of a consistent interpretation thereof, such an exploratory approach can be useful to shed light on potential mechanisms driving across-methods (in)stability.

Directly after a decision in any of the four tasks has been submitted, participants were asked to assess how risky they perceive their decision to be and how confident they feel about the particular choice they made. Each decision was depicted, as participants have just completed it, on a separate screen and questions were answered on a scale from 1 (“not at all risky/confident”) to 7 (“very risky/confident”). On the premise that subjects’ risk preferences are a stable trait, and that the four tasks elicit the same preference relation, one would expect to observe identical—or at least similar—assessments of the riskiness of choices across the four tasks on the individual level.

To examine whether insufficient comprehension of the elicitation procedures gives rise to increased across-methods variation in revealed risk preferences, the experimental protocol included comprehension questions and an eight-item Rasch-validated numeracy inventory (Weller et al. 2013). For the comprehension questions, subjects were shown a screenshot of the risk neutral decision in each of the four tasks, and were asked to estimate (1) the expected payoff, (2) the probability to earn less than €5.50, and (3) the probability to earn more than €14.50. Given the assumption that participants’ choices are dictated by some latent, deterministic preference relation, mistakes in evaluating the available lottery choices might impair across-methods consistency. We, thus, conjecture that the likelihood of making mistakes is negatively related to subject’s numeracy and comprehension of tasks. Accordingly, we expect to observe a negative relation between across-methods preference variation and comprehension and numeracy, respectively.

Moreover, we elicited several qualitative judgments on how subjects perceive the tasks relative to the other methods. After completing all elicitation methods, subjects were presented with additional questionnaires, requiring them to explicitly compare the four elicitation methods with regards to various dimensions on a single screen. In particular, we asked participants to evaluate each of the four elicitation methods with respect to (1) whether the instructions are easy to understand, (2) whether answering the task involves complex calculations, (3) whether the task is boring, and (4) whether the decision problem is associated with an investment, gambling, or insurance domain. Each of the questions (1) to (3) was answered on a scale from 1 (“not agree at all’) to 7 (“fully agree”). For answering question (4), subjects had to indicate one of the domains using a drop-down field. We hypothesize to find more noisy behavior within tasks that are perceived to be complex. Furthermore, subjects’ association with a specific domain serves as a means to examine whether revealed risk preferences are domain-specific. We conjecture to find less variation in revealed preferences for elicitation methods that are assigned to the same domain compared to elicitation methods that are associated with different domains.

## Analysis framework

For the analysis of the experimental data, we assume an expected utility theory (eut) framework. To estimate risk preferences, we assume a standard isoelastic utility function—a member of the family of power utility functions—of the form

1 $$ u(x) = {\left\{ \begin{array}{ll} (1-\varphi )^{-1}\ x^{1 - \varphi } &{}\text {if } \varphi \ne 1\\ ln(x) &{}\text {if } \varphi = 1 \end{array}\right. } $$

which is characterized by constant relative risk aversion (crra). This specification of utility curvature has been widely used in economics and related fields, and has been shown to typically better fit experimental data than alternative families (Camerer and Ho 1994; Wakker 2008).

In many within-subject experiments, the across-methods (in)stability of risk preferences is assessed based on correlations between the number of risky choices in different tasks. While significantly positive correlations might indicate that a certain degree of preference stability cannot be readily dismissed as spurious associations, correlations are actually not a conclusive measure (if a parametric utility function is assumed). Particularly, correlation coefficients measure the strength of the relationship between two variables—a characteristic that constitutes neither a necessary nor a sufficient condition for preference stability. In fact, it can be shown that choices in two tasks can be perfectly (rank order) correlated even if preferences vary dramatically between tasks; likewise, it can be shown that even perfectly stable preferences may result in (rank order) correlations of small magnitude.^7^ Therefore, the magnitude of correlations between the number of risky choices in two tasks cannot be readily interpreted as evidence in favor of or against the stability of risk preferences.

For this reason, we use another individual-level measure of across-methods stability of revealed preferences. Note that the assumption of a parametric functional form of a participant’s utility function implies that observed choices in a risk preference elicitation method translate into parameter intervals rather than point estimates. We define choices in two independent tasks as “stable” if the implied parameter intervals overlap (see, e.g., Bruner 2009). Whenever the sets of feasible parameters implied by the choices in two methods intersect, it cannot be ruled out that the observed choices do indeed stem from the same latent parameter $\varphi$. In particular, we define an indicator for each pairwise comparison of methods, which is equal to one if the implied parameter intervals overlap, and zero otherwise. As a preference stability index, we sum up these binary indicators for all six unique pairwise combinations of the four experimental risk preference elicitation methods, implying a measure between 0 and 6 on the individual level. This measure is conservative for two reasons: First, overlapping parameter intervals do not necessarily imply identical risk aversion parameters and, thus, across-methods stability of risk preferences. Second, overlapping parameter intervals could eventually be the result of random behavior or chance. For these reasons, the index has to be interpreted as a proxy for preference invariance.

In addition to the individual-level preference stability index we examine across-methods variation of risk preferences on the aggregate level by estimating a structural model for each elicitation method. We follow the procedure for structural model estimation for binary discrete choices under risk discussed in Harrison and Ruström (2008) and Wilcox (2008). Given the assumption of an eut framework, the probabilities $p_k$ for the high and low lottery payoffs $k \in \{h,l\}$ are those that are induced in the particular elicitation method by the experimenter. Thus, the expected utility of lottery $j \in \{A,B\}$, $E[u_j]$, is the utility of each lottery outcome, $u_k$, weighted by the corresponding probability:

2 $$ E[u_j] = \sum _{k} p_k u_k \quad \forall \ k \in \{h, l\} $$

For each of the $i = 1,2,\ldots ,n$ lottery pairs, participants are assumed to choose either the less risky (or safe) lottery $A_i$ or the more risky lottery $B_i$ by evaluating the difference between their expected utilities.^8^ In addition, we allow for mistakes or “tremble” in comparing the expected utilities of the alternatives participants face, modeled as a *Fechner* error term (see, e.g., Hey and Orme 1994; Loomes et al. 2002), yielding the latent index

3 $$ \nabla EU_i = E[u_{B_i}] - E[u_{A_i}] + \sigma \epsilon \quad \text { with } \epsilon \sim N(0, 1) $$

The additive component $\sigma \epsilon$ is a stochastic error term and can be interpreted as capturing noise in the decision maker’s evaluation of the difference between the lotteries’ expected utilities, with $\sigma$ being proportional to the standard deviation of this noise (Wilcox 2008).

The index $\nabla EU_i$, determined by latent preferences, is then linked to the participants’ observed choices using the cumulative standard normal distribution $\Phi (\cdot )$.^9^ This implies that the latent variable model of a considered choice probability using a probit link function is given by

4 $$ \begin{aligned} P(B_i \succ A_i)&= \Phi \left( \nabla EU_i \right) \\ P(B_i \succ A_i)&= \Phi \left( \sigma ^{-1} \left( E[u_{B_i}] - E[u_{A_i}]\right) \right) \end{aligned}$$

That is, the latent index $\nabla EU_i$ is linked to the observed choices by the specification that lottery $B_i$ is chosen whenever $\Phi (\nabla EU_i) > {1}/{2}$. As the standard deviation of the structural noise term, $\sigma$, approaches zero, the probability that the observed choice reflects the latent preference relation converges towards one.

The likelihood of participants’ responses, $L(\cdot )$, thus, is a function of the crra parameter $\varphi$, the standard deviation of the structural noise $\sigma$, and the vector of *n* choices observed in the experimental task ($\vec {y}$). The conditional log-likelihood function is given by

5 $$ \ln L(\varphi , \sigma | \vec {y}) = \sum _{i = 1}^{n} \bigg ( \Big [ \ln \Phi \big ( \nabla E[u_i] \big ) \Big ]^{y_i} + \Big [ \ln \Phi \big ( -\nabla E[u_i] \big ) \Big ]^{1 - y_i} \bigg ) $$

where $y_i$ denotes an indicator function taking value 1 if a participant chooses the more risky lottery $B_i$ and zero otherwise, for all $i = 1, 2, \ldots , n$. The function $\ln L(\varphi , \sigma | \vec {y})$ is maximized with respect to $\varphi$ and $\sigma$, with standard errors being clustered on the subject level, reproducing the routines for *Stata* proposed by Harrison and Ruström (2008).

At this point it should be noted that random utility models, such as the model delineated above, have recently been shown to be prone to violations of monotonicity. In particular, the choice probability $P(B_i \succ A_i)$ is not necessarily a decreasing function of the crra parameter $\varphi$, whereas random parameter models are always monotone in this regard (Apesteguia and Ballester 2018). However, in our setting, the methodology of the random parameter model has disadvantages—in particular, a loss of observations (see “Appendix 6” for details in Electronic Supplementary Material). As argued by Apesteguia and Ballester (2018), the practical implications of monotonicity violations are twofold: (1) The use of random utility models may pose identification problems since the same choice probabilities may be associated with different levels of risk aversion; and (2) there might be an upper limit to the level of risk aversion if subjects are extremely risk averse. While (1) turns out not to apply to random utility model estimates for the four risk preference elicitation tasks included in our experiment, (2) is unlikely to pose problems in aggregate level estimates for our sample, as the share of extremely risk averse subjects is very low. Moreover, our main analysis relates to the *relative*, rather than the absolute, magnitude of risk aversion estimates. Overall, we consider the drawbacks in utilizing the random parameter model to loom larger than the bias resulting from *potential* violations of monotonicity in the random utility model. For this reason, we assume a random utility model in our analysis and only refer to the alternative model specification where relevant.

## Results

In what follows, we first present evidence on the across-methods heterogeneity of revealed risk preferences, then relate it to subjects’ perceived riskiness of choices, and finally discuss implications and potential explanations of our findings in the light of the related literature.

### Across-methods variability of revealed risk preferences

In line with previous results on across-methods variation in risk preferences (see, e.g., Deck et al. 2013; Dulleck et al. 2015; Csermely and Rabas 2016; Pedroni et al. 2017, we find that Spearman rank correlations between the observed number of risky choices in the four tasks are moderate but significantly different from zero, varying between 0.222 and 0.367; polychoric correlations are slightly higher and vary between 0.245 and 0.400 (Table 1). Only 71.7% of the participants are consistently risk averse in all four tasks. For the remaining 28.3% of the participants, choices are associated with risk loving preferences at least once. However, the significantly positive pairwise correlations indicate that more risky choices in one task, on average, are associated with more risky choices in another task.

**Table 1:** Correlation matrix. The lower triangular matrix reports Spearman rank correlations between the observed number of risky choices in the four tasks; the upper triangular matrix depicts polychoric correlations

	bret	cem	mpl	scl
bret		0.245	0.350	0.336
		(0.001)	(0.000)	(0.000)
cem	0.222		0.283	0.400
	(0.002)		(0.000)	(0.000)
mpl	0.367	0.244		0.387
	(0.000)	(0.001)		(0.000)
scl	0.341	0.338	0.354	
	(0.000)	(0.000)	(0.000)	

*p* values are reported in parentheses ($n = 198$). bret, cem, mpl, and scl denote the “bomb” risk elicitation task, the certainty equivalent method, the multiple price list, and the single choice list, respectively

Turning towards our preference stability index, subjects on average reveal stable risk preferences in 2.8 ($sd = 1.5$) out of 6 possible combinations.^10^ In order to appropriately interpret the degree of observed variation in preferences, it is informative to relate the experimental data to sensible benchmarks. The theoretical upper bound of the preference stability index is derived from a hypothetical subject with deterministic and stable preferences who does not make any mistakes in revealing her preferences in any of the tasks. Such a subject would act exactly as her $\varphi$ dictates and reveal invariant preferences in all six pairwise comparisons in our setting.

As the sets of feasible crra interval estimates implied by participants’ choices in the elicitation methods might intersect by pure chance, even random behavior can be expected to manifest itself in a preference stability index larger than zero. To approximate a lower benchmark, we thus simulate uniformly distributed choices for each of the four methods for 10,000 virtual subjects characterized by the preference functional as described above. Indeed, these simulations reveal that the lower benchmark is substantially larger than zero ($m = 1.3$, $sd = 1.1$), with only $\sim {1}/{4}$ of the simulation outcomes ending up with 0 out of 6 possible intersections of crra point estimate sets. Two more simulation exercises are informative as benchmarks for the experimental data. In the first simulation, choices for each of the four tasks are drawn *independently* from the choice distribution observed in the experimental data. By this means, the simulation exercise assumes that subjects treat each of the tasks independently. An alternative benchmark, motivated by Crosetto and Filippin (2015), is determined by virtual subjects exhibiting stochastic preferences. For this purpose, we simulate another 10,000 virtual subjects characterized by some latent crra parameter $\varphi _l$ but add some i.i.d. noise directly to subject’s inherent risk preferences for each of the four methods. In particular, we assume that the virtual subjects’ latent parameter $\varphi _l$ is normally distributed, with $\mu _l = 0.6$ and $\sigma _l = 0.3$. That is, the actual $\varphi _a$ determining virtual subject’s choices departs from their real, latent $\varphi _l$ by some stochastic noise with zero mean and standard deviation $\sigma _a$, i.e., $\varphi _a = \varphi _l + \sigma _a, \sigma _a \sim N(0, 0.3)$.

**Fig. 1:**
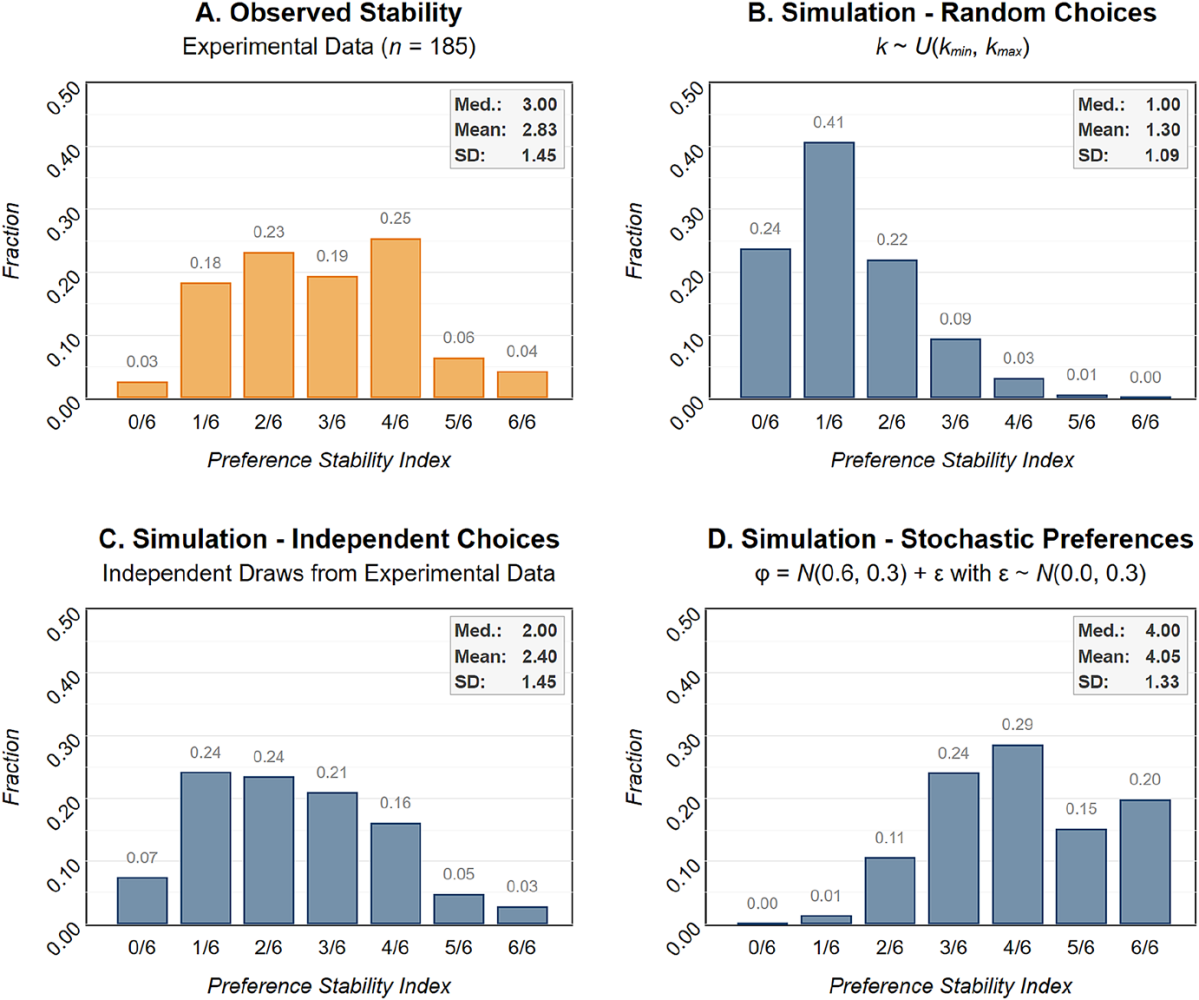
**a** Distribution of the preference stability index (number of pairwise comparisons in which implied parameter intervals overlap) for the experimental data ($n = 185$). **b** Simulation exercise with virtual subjects choosing uniformly and independently from the available choices in each of the four risk preference elicitation methods. **c** Simulation exercise with virtual subjects choosing independently from the choice distribution of each task observed in the experiment. **d** Simulation exercise with virtual subjects with stochastic preferences, where a noise term $\epsilon \sim N(0, 0.3)$ is added directly to subjects’ crra parameter $\varphi \sim N(0.6, 0.3)$. $n = 10,000$ for each simulation

The distributions of the preference stability index observed in the experiment as well as the results of the three simulations are depicted in Fig. 1. Eyeballing the histograms indicates that the distribution from the experimental data (Panel A) can neither be fully explained by subjects choosing uniformly at random (Panel B), nor by subjects characterized by stochastic preferences (Panel D). While the simulation of random choices constitute a lower benchmark and expectedly results in a right-skewed distribution of the preference stability index, the stochastic preferences assumptions imply a distinctly left-skewed distribution. The simulation outcomes of independent draws from the experimental data (Panel C), however, highlight considerable similarities to the experimental data. This is a surprising result, as the observed distribution in the experiment reveals a behavioral pattern that appears as if subjects would choose *independently* across the four elicitation methods.^11^ This observation immediately raises the question *why* participants exhibit such a high level of variation in revealed risk preferences.^12^

### Perceived riskiness of choices

On the aggregate level, we estimate structural models for each of the tasks, as described in Sect. 4. The corresponding maximum likelihood estimates, ${\hat{\varphi }}$ and ${\hat{\sigma }}$, are reported in Table 2A. Estimates of both the crra coefficient and the variance of noise vary substantially across the four risk preference elicitation tasks. The crra estimates are significantly different from one another for all pairwise comparisons of methods, except for ${\hat{\varphi }}_{\textsc {bret}}$ and ${\hat{\varphi }}_{\textsc {mpl}}$ (lower triangular matrix in Table 2B); the differences between the estimates of the variance of the structural noise term are statistically significant for all comparisons of methods (upper triangular matrix in Table 2B). Note that the maximum likelihood estimates of the crra parameter $\varphi$ are comparable to estimates reported in the literature in terms of magnitude. In particular, we are not the first to report that subjects, on average, tend to be significantly more risk averse in the bret and the mpl than in the scl (see, e.g., Dave et al. 2010; Crosetto and Filippin 2015).

**Table 2:** **(A)** Maximum likelihood estimates of structural models with Fechner error terms for each of the four risk preference elicitation methods. Standard errors, clustered on the subject level, are reported in parentheses. **(B)** Pairwise differences in point estimates of risk preference parameters $\varphi$ (lower-triangular matrix) and the standard deviation of noise parameters $\sigma$ (upper-triangular matrix) between the four risk preference elicitation methods

	bret	cem	mpl	scl
*Panel A*				
$\varphi$	0.626***	0.838***	0.602***	0.387***
	(0.021)	(0.090)	(0.033)	(0.034)
$\sigma$	0.046***	0.263***	0.977***	0.720***
	(0.002)	(0.048)	(0.066)	(0.057)
$\ln L$	− 5,298	− 458	− 600	− 572
No. of Obs.	19,800	1782	1980	990
Clusters	198	198	198	198
*Panel B*				
bret		− 0.217***	− 0.932***	− 0.674***
cem	0.212*		− 0.715***	− 0.457***
mpl	− 0.025	− 0.237**		0.257**
scl	− 0.240***	− 0.452***	− 0.215***	

*p* values are based on pairwise Wald tests. bret, cem, mpl, and scl denote the “bomb” risk elicitation task, the certainty equivalent method, the multiple price list, and the single choice list, respectively. *$p<0.05$, **$p<0.01$, ***$p<0.001$

**Fig. 2:**
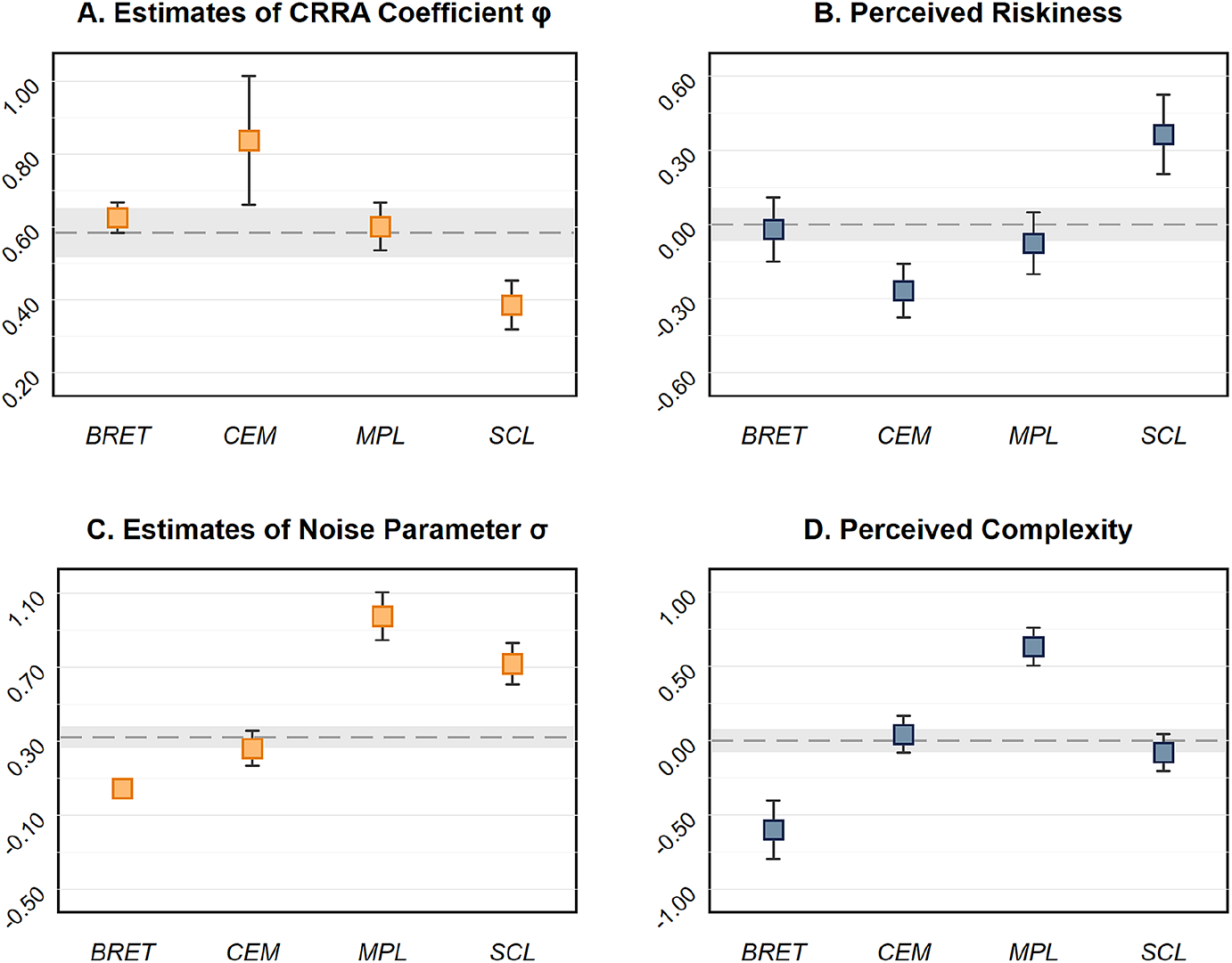
**a** Maximum likelihood estimates of crra coefficients $\varphi$. **b** Average perceived riskiness (subject-demeaned data) for the four risk preference elicitation methods. **c** Maximum likelihood estimates of the standard deviation of the structural noise parameter $\sigma$. **d** Average perceived complexity (subject-demeaned data) for the four risk preference elicitation methods. In all panels, error bars indicate 95% confidence intervals. The dashed lines indicate the overall estimate (pooling all tasks) in Panels **a** and **c** (${\hat{\varphi }} = 0.585$ and ${\hat{\sigma }} = 0.324$), and depict means in Panels **b** and **d**; shaded areas indicate 95% confidence intervals. Standard errors in the maximum likelihood estimations are clustered on the individual level; $n = 198$. bret, cem, mpl, and scl denote the “bomb” risk elicitation task, the certainty equivalent method, the multiple price list, and the single choice list, respectively

Comparing crra point estimates ${\hat{\varphi }}$ (Fig. 2a) to the average subject-level demeaned perceived riskiness of each task (Fig. 2b) reveals a remarkable result. Not only do the assessments of riskiness differ considerably across tasks, but the almost perfectly mirrored patterns suggest that, on average, subjects are well aware of the level of and the across-methods variation in the riskiness associated with their choices. This is a strong indicator that subjects *deliberately* take different levels of risk across tasks.^13^ This awareness even extends to the participants’ assessment of the difficulty of tasks. Panels C and D of Fig. 2 depict maximum likelihood estimates of the standard deviation of the noise parameter $\sigma$ in the structural model for each elicitation method as well as the average subject-level demeaned perception of the tasks’ complexity. Again, both patterns look similar to a remarkable extent, indicating that subjects, on average, can well assess the susceptibility to mistakes or “trembles” in revealing their actual preferences across methods.^14^

We provide additional evidence on subjects’ awareness of varying levels of risk associated with seemingly inconsistent choices across methods by extending the structural model specification outlined in Sect. 4. In particular, we estimate ${\hat{\varphi }} = {\hat{\varphi }}_0 + {\hat{\varphi }}_r \cdot r_p$ and ${\hat{\sigma }} = {\hat{\sigma }}_0 + {\hat{\sigma }}_c \cdot c_p$, where ${\hat{\varphi }}_0$ and ${\hat{\sigma }}_0$ are estimates of the constants and $r_p$ and $c_p$ refer to perceived (subject-level demeaned) riskiness and complexity, respectively. The maximum likelihood estimates of this model indicates that risk aversion is significantly related to participants’ evaluation of the choice’s riskiness (${\hat{\varphi }}_r = -0.131$, $p < 0.001$), and that the variance of the structural noise term significantly varies depending on subjects’ appraisal of task complexity (${\hat{\sigma }}_c = 0.065$, $p < 0.001$). Overall, our results indicate that subjects seem to be well aware of the riskiness of their choices as well as the complexity of the decision situation.

Our findings are in line with the observed zero correlation of (1) numeracy and (2) task comprehension with the preference stability index in our experimental data: We hypothesized that subjects’ ability to reveal their risk preferences may vary across the different elicitation methods. Subjects might make mistakes in evaluating the lotteries that are explicitly and implicitly contained in the elicitation procedures, and thus in correctly choosing the lotteries that match their preferences. Accordingly, we should find a significant correlation between subjects’ level of preference stability and (1) the absolute difference between the responses and the correct answers to the comprehension questions,^15^ and (2) the achieved numeracy score. However, both correlations are low and insignificant ($\varrho =-0.089, p=0.210$ and $\varrho =0.033, p=0.649$, respectively). Thus, we do not find evidence of a positive relation between a subject’s numeracy or comprehension of tasks and the degree of preference stability across tasks.^16^ We deem this finding anything but trivial. It supports the basic assumption that risk preference elicitation methods are indeed designed in a way that subjects are able to reveal their preferences irrespective of their explicit understanding of the calculations behind the lotteries. Moreover, these zero correlations are in line with our conclusion that subjects are well aware of the difficulty of methods and the susceptibility to mistakes, but still make choices that differ in riskiness across tasks.

How do our findings relate to the procedural invariance axiom, preference (in)stability, and the interpretation of (in)consistency? As argued above, the validity of the assumptions of preference stability and procedural invariance—both of which are the premises for the interpretation of inconsistency—cannot be assessed independently of one another. Yet, we argue that our findings cannot be readily reconciled with the joint assumption of preference stability and procedural invariance, which casts doubt on interpreting across-methods variation in reveal preferences as inconsistent behavior. Particularly, the result that subjects are aware of how much risk they take challenges the interpretation of inconsistency. For the sake of the argument let us assume that participants have stable risk preferences *and* that the four tasks in our experiment indeed elicit the same preference relation, i.e., that the procedural invariance axiom holds. Given these two assumptions, there are two possibilities for subjects to behave inconsistently in our experiment: First, participants could be *unaware* of the across-methods variation in their risk-taking behavior. This kind of unawareness, however, is not in line with our data, since unaware subjects with stable risk preferences would have to consider their decisions in each method equally risky. Second, subjects could be well *aware* of the variation in their risk-taking behavior. In our experiment, the systematic differences in risk perception across methods indicate subjects’ awareness of the *systematic* variation in revealed preferences. There is no reason to believe that subjects systematically and deliberately decide contrary to their actual preference relations, which are assumed to be stable and invariantly measured by the various methods. Thus, we argue that our findings cannot be readily reconciled with the interpretation of inconsistency.

One potential explanation of the variation in risk attitudes across methods is to discard the procedural invariance axiom in exchange for the assumption that subjects have domain-specific risk preferences for different types of choices (Weber et al. 2002). To account for this possibility, we elicited subjects’ association of methods with an investment, gambling, or insurance domain. For pairwise comparisons of methods, we test if the preference stability index is higher for subjects that assign the same domain to the two tasks compared. As reported in Table 4 in “Appendix 3” in Electronic Supplementary Material, we do not find a significant effect for any of the pairwise comparisons. Thus, we cannot conclude that domain-specificity explains the observed variation in revealed risk preferences in our data. Although our measure of domain-specificity, with only three choice-options for associated domains, is rather crude, our result is in line with previous findings (see, e.g., Deck et al. 2013). Given that our choice of domains is motivated by real-world contexts, i.e., investment, gambling, and insurance, our finding also relates to recent evidence that calls into question the external validity of experimental measures of risk preferences (see Charness et al. 2019).^17^

## Summary and discussion

We conduct a within-subjects experiment with 198 participants, examining the heterogeneity in revealed risk preferences across four different, widely used risk preference elicitation tasks. In line with previous studies, we find substantial variation in revealed risk preferences. While earlier studies usually assess the across-methods variation using correlations between risky choices in the different tasks, we discuss drawbacks of this approach and introduce an individual-level measure that is based on whether or not the implied crra parameter intervals overlap. Based on this measure we report that subjects’ risk preferences, on average, are stable in less than half of the pairwise comparisons of methods. Comparing the observed behavior to results from simulation exercises, we find that the observed heterogeneity in risk preferences across tasks is qualitatively similar to the heterogeneity arising from independent random draws from the choices in the experiment. As such, our study adds a novel perspective to the “risk elicitation puzzle” by quantifying the degree of the variability of preferences across methods by use of an alternative measure, benchmarked to the results of agent-based simulations. Yet, the primary goal of our paper is to contribute to the *understanding* of regularly reported across-method variation in risk preferences. As an innovative contribution, we relate the observed behavior to subjects’ perceived riskiness of choices reported in a questionnaire. Notably, we find that subjects are well aware of the level of risk associated with their decisions, even though the observed behavior can be characterized by varying risk attitudes. We interpret this as a piece of evidence that participants make their choices *deliberately* and argue that this suggests that subjects’ behavior cannot be readily interpreted as inconsistent. In particular, interpreting the variation in revealed risk preferences as inconsistent involves the assumptions of both preference stability and procedural invariance. Since our data suggests that subjects are aware of the *systematic* across-methods variation in their choices, the heterogeneity in revealed risk preferences can only be reconciled with the interpretation of inconsistency if one accepts that participants systematically and deliberately decide contrary to their actual preference relations. We deem this interpretation implausible and, thus, argue that the common assumption of procedural invariance and across-methods stability of preferences should be reconsidered. Yet, it is not clear which of the two premises—the procedural invariance axiom or the assumption of preference stability (or both) – is refuted by our results, since the validity of either of the two presumptions cannot be separately inferred from the observation of across-methods heterogeneity of preferences. We believe that it is a significant challenge for future research to find a way to empirically disentangle the two concepts and test them in isolation.

While our study adds a novel perspective to a hotly debated topic in experimental economics, potential limitations should be considered when interpreting our findings. Our experimental design is not equipped to test whether certain characteristics of the elicitation methods might affect behavior in a way that could lead to the observed heterogeneity in revealed risk preferences. For instance, it has been argued that the choice structure of tasks might impact participants’ risk-taking behavior. Examples are provided by Andersen et al. (2006), showing that the available lotteries affect choices, and by Crosetto and Filippin (2017), showing that the omission of alternatives influences risk-taking. Relatedly, He and Hong (2017) illustrate that subjects tend to make less risky decisions in a choice environment that is perceived as more risky. Risk-taking behavior, for instance, might be influenced by the worst possible outcome in the task (Anzoni and Zeisberger 2016; Holzmeister et al. 2020). More generally, Vosgerau and Peer (2018) provide evidence for the malleability of preferences under uncertainty. Moreover, Carbone and Hey (1995) argue that the preference functional that can explain subjects’ choices may be conditional on the elicitation method. The availability of a focal safe alternative, for example, might affect subjects’ choice behavior. As argued by Crosetto and Filippin (2015), a safe option could serve as a reference point against which outcomes are evaluated, potentially inducing failures of Expected Utility Theory (see e.g., Andreoni and Sprenger 2012; Camerer 1992; Starmer 2000. Generally speaking, Expected Utility Theory might not be the most appropriate framework to model subjects’ preferences. Rather, participants might have reference point-dependent preferences, comprising loss, regret, or disappointment aversion (see, e.g., Kahneman and Tversky 1979; Loomes and Sugden 1982; Gul 1991). However, Zhou and Hey (2017) suggest that the elicitation of risk attitudes is more sensible to the method used than the assumed preference functional. In line with these results, Pedroni et al. (2017) and Friedman et al. (2018) do not find evidence for superior alternative explanatory frameworks. Although our study does not provide conclusive insights into these matters, we hope that our finding help to identify promising avenues for future research.

Our results shed light on previous findings on within- as well as between-subject variation of revealed risk preferences across different elicitation methods, in that observed behavior might not be easily dismissed as inconsistent. This calls for a reassessment of the common research practice of choosing among different elicitation procedures based on purely pragmatic reasons. Our findings indicate that the choice of the elicitation method may well have a major impact on the elicited preferences. The results reported in this paper should serve as an invitation to reconsider and reassess the assumptions of procedural invariance of methods and preference stability, as well as the interpretation of inconsistency. Eventually, we hope that our study contributes to a fruitful discussion on the across-methods variability of risk preferences and the methodology of preference elicitation in general.

## Electronic supplementary material

Below is the link to the electronic supplementary material.

Supplementary material 1 (pdf 2441 KB)

## Footnotes

1 In our paper, for the sake of conceptual clarity, we use the term “preference (in)stability” to describe a subject’s underlying risk preference trait, whereas we use the term “inconsistency” to describe behavior that is contradictory given a subject’s preference relation. We might deviate from the literature in this respect.

2 Please note that our outline of the related literature comprises results from the economic and the psychological literature alike. While the two fields may differ in their methodological approaches, e.g., regarding the focus on normative aspects of preference elicitation or the external validity of different measures, we deem these distinctions of secondary importance for a summary of the evidence on seemingly inconsistent behavior in incentivized risk preference elicitation tasks.

3 Note that, despite a random sequence of tasks, the order in which subjects face the elicitation methods might affect their choices. Thus, we provide a comprehensive analysis of potential order effects in “Appendix 5” in Electronic Supplementary Material. The results are not indicative of any systematic effects attributable to the task ordering.

4 Examining the stability of risk preferences across different methods *on the individual level* calls for a within-subjects experimental design. A within-subject design may induce cross-task contamination effects and necessitates the random lottery incentive system, which effectively introduces a compound lottery. Starmer and Sugden (1991); Cubitt et al. (1998) provide empirical evidence for the validity of the random lottery incentive system and do not find an indication of contamination effects (see also Harrison and Ruström 2008). In line with these results, our analysis of potential order effects (reported in detail in “Appendix 5” in Electronic Supplementary Material) does not point towards contaminating effects between tasks in our data.

5 Note that by enforcing a single switching point, we impose that subjects comply with monotonicity and transitivity requirements, foregoing any opportunity to check whether this is actually the case. Apart from enforcing a single switching point, several alternatives how to deal with multiple switching behavior have been proposed in the literature, such as dropping observations (e.g., Deck et al. 2013), treating the number of safe choices as an indicator of risk preferences (e.g., Holt and Laury 2002), or adding an indifference option to the choice list (e.g., Andersen et al. 2006).

6 In Crosetto and Filippin (2013)’s baseline condition “Dynamic,” boxes are not collected randomly but sequentially. Our implementation corresponds to their robustness treatment “Random.” While the mean number of boxes collected in the “Random” condition is slightly smaller than in the baseline treatment “Dynamic,” the distribution of choices across the two treatments does not differ significantly.

7 For the sake of illustration, consider the following examples: (1) Suppose half of the subjects in an hypothetical experiment chooses 60 boxes in the bret and lottery $L_3$ in the scl; suppose the other half chooses 70 boxes in the bret and lottery $L_4$ in the scl. Apparently, the (rank order) correlation coefficient between the choices in the two tasks would be + 1, even though all subjects reveal to be risk-loving in the bret but risk averse in the scl. (2) Consider subjects’ choices in, e.g., the cem and the mpl. Suppose there are three types of subjects, characterized by the crra parameters $\varphi _1 = 1.10$, $\varphi _2 = 0.95$, and $\varphi _3 = 0.80$, and assume that subjects’ choices are solely dictated by their crra parameter without error. Then, in the cem, types $\varphi _1$ and $\varphi _2$ will choose the risky lottery three times, whereas type $\varphi _3$ prefers the lottery four times. In the mpl, type $\varphi _1$ will prefer the more risky alternative two times, whereas types $\varphi _2$ and $\varphi _3$ will choose the more risky lottery three times. If ${(n-1)}/{2}$ subjects are of type $\varphi _1$, ${(n-1)}/{2}$ are of type $\varphi _2$, and one subject is of type $\varphi _3$, the rank order correlation between the number of risky choices will converge to zero as $n \rightarrow \infty$. In general, whenever the parameter intervals implied by the choices in the two elicitation methods do not exactly coincide, the magnitude of (rank order) correlations between the choices in two tasks may be considerably smaller than 1, even if preferences are stable across tasks.

8 In order to apply this procedure, choices in all elicitation methods need to be expressed as a series of binary choices between lottery pairs. While this is the case for the cem and the mpl by default, data from the bret and the scl need to be transformed. Following Dave et al. (2010) and Crosetto and Filippin (2015), we convert the gambles in bret and scl into implicit binary choices between two adjacent gambles assuming that utility functions are well-behaved, i.e., that preferences are single-peaked. Thus, for the bret, for instance, a subject selecting 40 out of 100 boxes is assumed not only to reveal that 40 boxes are preferred to 39 but also that 39 boxes are preferred to 38, 40 boxes are preferred to 41, etc. The same rationale is applied to the observed choices in the scl.

9 Alternatively, the probit link could be replaced by a logit link as proposed by Luce and Suppes (1965), and employed by Camerer and Ho (1994) and Dave et al. (2010) among others. For our data, the results turn out to be qualitatively akin for either of the two functional specifications.

10 bret, mpl, and cem include at least one first-order dominated choice each. Of the 198 subjects in our sample, 13 (6.6%) violate basic rationality by choosing a dominated lottery in at least one of the tasks: 1 (0.5%) in bret, 6 (3.0%) in cem, and 9 (4.5%) in mpl. As dominated choices cannot be reasonably translated into crra intervals, the preference stability index cannot be determined for participants violating rationality. Thus, any result referring to the preference stability index is based on the sample with $n = 185$.

11 Examining whether the distributions depicted in Panels A and C of Fig. 1 differ significantly requires some consideration. In short, to allow for an unbiased comparison, we chose a bootstrapping approach (10,000 iterations) with equal sample sizes. Kolmogorov–Smirnov tests suggest that the distributions do *not* significantly differ in 70% of the cases. For a thorough outline of our approach and a discussion of this result, please refer to “Appendix 4” in Electronic Supplementary Material.

12 Distinct mechanics of the tasks—such as the number of choices, their mapping into crra parameter intervals, or the range of the codomain—might have an effect on a task’s relative contribution to the preference stability index. For this reason, as a robustness check, we examine the preference stability index on a per-task basis in “Appendix 4” in Electronic Supplementary Material. While our experimental design does not allow to infer whether the identified differences can be attributed to task mechanics, we find that all tasks contribute to the overall index and that heterogeneity of individual risk preferences can also be found on the per-task level.

13 The result on deliberate risk-taking is well compatible with the finding in Dulleck et al. (2015), that only about 13% of participants want to change their decision when given the chance to do so.

14 It is reassuring that the estimates of $\varphi$ based on a random parameter model, reported in Table 7 in “Appendix 6” in Electronic Supplementary Material, are qualitatively similar to the results of the random utility model reported in Table 2. In particular, the ordering of point estimates for each of the four tasks is preserved, and the patterns of statistically significant differences remain similar using the alternative model specification.

15 For each of the three questions per task, we first calculate the absolute difference between a subject’s responses and the correct answers. In a second step we relate each deviation to the correct answer and average them on the subject level. For a comparison of relative absolute deviations per task see “Appendix 1” in Electronic Supplementary Material.

16 This is in line with previous literature, such as Pedroni et al. (2017). See also Andersson et al. (2016) and Andersson et al. (2018), who find that cognitive ability is related to noisy behavior rather than to risk preferences.

17 However, for supporting evidence on the external explanatory power of incentivized measures see, e.g., Lusk and Coble (2005) and Anderson and Mellor (2008); for survey based measures see, e.g., Barsky et al. (1997), Dohmen et al. (2011) and Beauchamp et al. (2017).
