# Supplementary material for: The risk elicitation puzzle revisited: Across-methods (in)consistency?
Source: Exp Econ. 2020 Sep 12;24(2):593–616. doi: 10.1007/s10683-020-09674-8 (PMC8550567; doi:10.1007/s10683-020-09674-8)
Supplement: Supplementary file 1 — Supplementary material 1 (pdf 2441 KB) [file 10683_2020_9674_MOESM1_ESM.pdf]

Electronic Supplementary Material

The risk elicitation puzzle revisited: Across-methods (in)consistency?

Felix Holzmeister[†,*] Matthias Stefan[‡]

[†] Department of Economics, University of Innsbruck
[‡] Department of Banking and Finance, University of Innsbruck

[*] Corresponding author: felix.holzmeister@uibk.ac.at

Contents

Appendix 1. Perception of Task Characteristics 2
Appendix 2. Task Comprehension and Numeracy 5
Appendix 3. Domain Attribution 6
Appendix 4. Preference Stability Index 7
Appendix 5. Order Effects 10
Appendix 6. Random Parameter Model 13
Appendix 7. Screenshots of the Experiment 15

Appendix 1. Perception of Task Characteristics

In order to investigate individual-level characteristics that could potentially explain the observed variability in revealed risk preferences across the four risk preference elicitation tasks, subjects in the experiment were asked to answer additional questionnaires. For each participant, to avoid confusion, specific questions on the tasks followed the same ordering in which the tasks were completed. To simplify discrimination between the four different decision problems, the tasks were labeled with color names and highlighted in the respective color whenever displayed to subjects (see Appendix 7 for screenshots of the entire experiment).

Immediately after subjects had made their decision in any of the four tasks, they self-reported how risky they perceive their decision to be and how confident they feel with the particular choice(s) they made. Each decision, as participants have just completed it, was depicted on screen and questions were answered on a scale from 1 ("not at all risky/confident") to 7 ("very risky/confident"): *(i)* "How risky do you consider your own decision (indicated above)?" and *(ii)* "How confident do you feel with your decision indicated above?" Experimental results of the answers to these questions are reported in Panels A and B of Fig. 3.

After completing all elicitation methods, participants answered additional questionnaires explicitly comparing the four tasks. For completing the comparative questionnaires, subjects received a payment of €3.00. Questions were answered on a scale from 1 ("not agree at all") to 7 ("fully agree") and read as follows: *(i)* "the task is easy to understand and can be answered straightforwardly," *(ii)* "the task involves complex calculations and requires deliberating on the trade-off between expected outcomes and the inherent riskiness of the different outcomes," and *(iii)* "completing the task is annoying and boring." Experimental results of the answers to these questions are reported in Panels C–E of Fig. 3, respectively. Tab. 3 reports correlations between the questionnaire items and the preference stability index.

Table 3: Correlations of the preference stability index (*PSI*) and responses to the questionnaire items. The lower triangular matrix depicts Spearman rank correlations; the upper triangular matrix reports polychoric correlations. p-values are reported in parentheses ($n = 198$). * $p < 0.05$, ** $p < 0.01$, *** $p < 0.001$.

	PSI	Risk.	Conf.	Simp.	Comp.	Bore.
PSI		0.127***	0.025	−0.050	0.053	0.049
		(0.001)	(0.502)	(0.231)	(0.163)	(0.273)
Riskiness	0.143***		−0.028	−0.015	0.093*	0.080
	(0.000)		(0.464)	(0.713)	(0.014)	(0.072)
Confidence	0.023	−0.025		0.191***	−0.005	−0.076
	(0.516)	(0.481)		(0.000)	(0.906)	(0.086)
Simplicity	−0.048	−0.038	0.156***		−0.394***	−0.177***
	(0.179)	(0.281)	(0.000)		(0.000)	(0.000)
Complexity	0.076*	0.099**	−0.008	−0.358***		0.127**
	(0.032)	(0.006)	(0.829)	(0.000)		(0.004)
Boredom	0.032	0.078*	−0.045	−0.129***	0.107**	
	(0.372)	(0.028)	(0.206)	(0.000)	(0.003)	

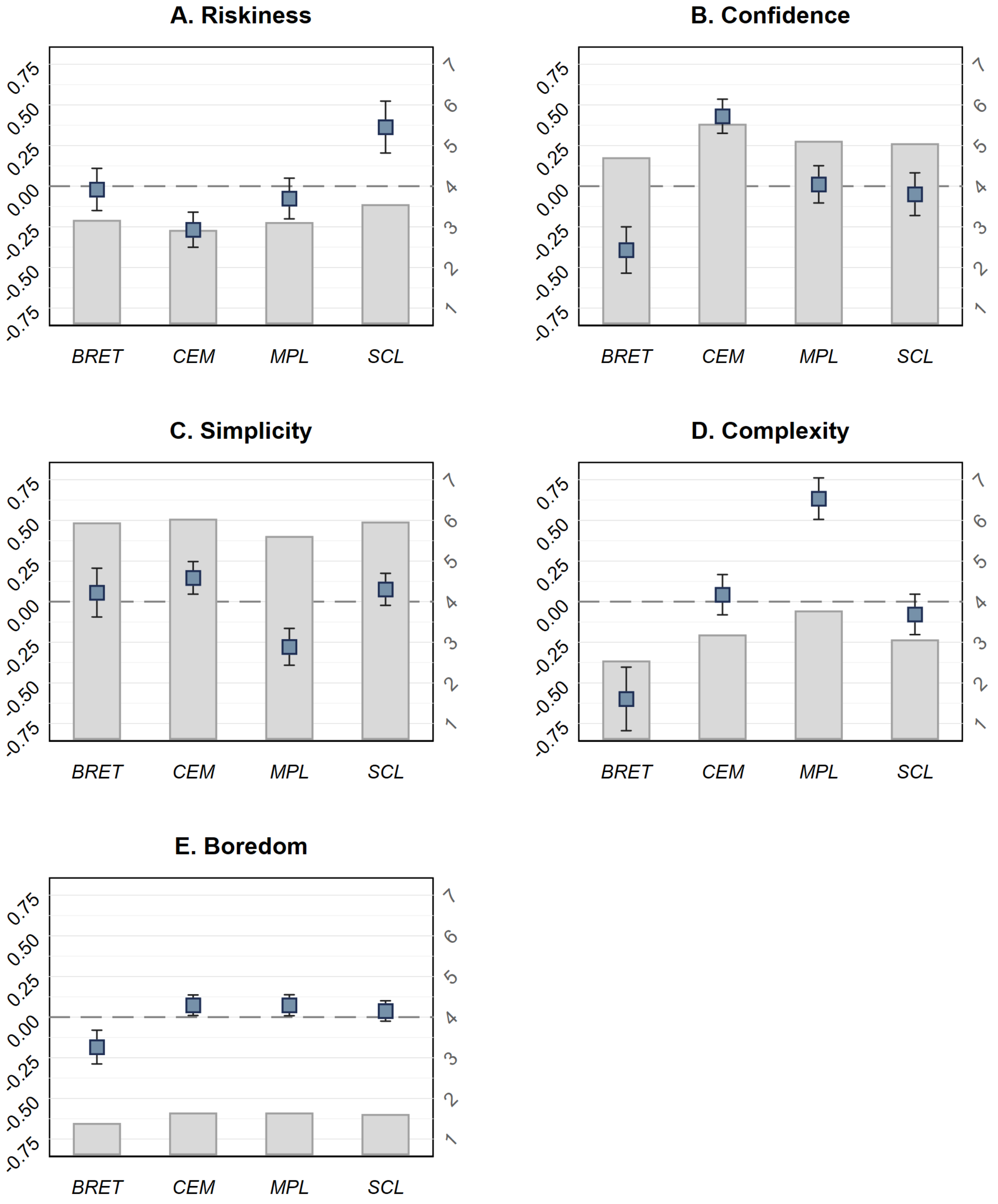

Figure 3: Subject-level demeaned scores (left y-axis) and mean levels (right y-axis) for self-reported answers to survey questions on (A) riskiness of own decision, (B) confidence in own decision, (C) simplicity of task instructions, (D) complexity of calculations involved, and (E) boredom, separated by tasks. In all panels, error bars indicate 95% confidence intervals; $n = 198$. BRET, CEM, MPL, and SCL denote the "bomb" risk elicitation task, the certainty equivalent method, the multiple price list, and the single choice list, respectively.

Several characteristics in subjects' responses to the five questions as depicted in Fig. 3 seem noteworthy: First, general levels of mean responses on confidence and simplicity of choices across tasks are fairly high, but rather low for answers on boredom and complexity. In general, this can be considered as good news for experimental research on risk preferences. Second, there is substantial variation in response levels of riskiness, confidence, simplicity, and complexity. Thus, subjects seem to perceive the tasks

and their choices across methods quite differently. In addition to the findings reported in the paper, this result calls for more caution in choosing a particular method to elicit risk preferences. Third, while perceived complexity of the tasks seems to clearly relate to subjects' mistakes, as reported in the paper, self-assessed confidence on a subject's decision does not. We conjecture that the assessed confidence does encompass a variety of subjects' attributes, rather than only relating to complexity and difficulty of methods.

At the very end of the experiment, participants were asked to state their preferences for a task in future experiments (as a single choice). Of the 198 subjects, 30.8% prefer the BRET, 31.3% the CEM, 21.7% the MPL, and 16.2% the SCL.

Appendix 2. Task Comprehension and Numeracy

Subjects were asked to estimate (i) the expected payoff, (ii) the probability to earn less than €5.50, and (iii) the probability to earn more than €14.50 for the risk neutral decision (depicted as a screenshot) in each of the four tasks. On average, subjects' responses deviate from the correct answers by 164.4% (sd = 92.4%) in the BRET, 111.7% (sd = 69.7%) in the CEM, 177.0% (sd = 95.6%) in the MPL, and 57.7% (sd = 60.9%) in the SCL.

In addition, to assess participants' numerical skills, we included an eight-item Rasch-validated numeracy inventory (Weller et al., 2013), including two items on cognitive reflection.[1] The numeracy inventory was incentivized with €0.50 for each correct answer. On average, participants correctly answered 5.49 ($sd = 1.57$) out of 8 questions.

While subjects have rather high levels of numeracy, their estimation of expected returns and probabilities show strong deviations from the correct answers. Furthermore, it is noteworthy that actual errors in estimations are not necessarily in line with the perceived complexity of tasks. While subjects seem to be able to assess the susceptibility to errors in making choices in the tasks, as argued in the paper, this self-assessment seems not to be directly related to the ability to calculate expected returns and probabilities. This supports the—often implicitly made—assumption that subjects can reveal their preferences in the tasks without explicitly being able to correctly solve the calculations behind the tasks' lotteries.

[1] The inventory proposed by Weller et al. (2013) includes two of the three cognitive reflection test items introduced by Frederick (2005). As these questions have been used repeatedly in our laboratory and correct answers to the questions might be known, we replaced these items by two questions proposed by Toplak et al. (2014).

Appendix 3. Domain Attribution

In comparing the four elicitation methods, subjects were also asked whether they associate the decision problem with an investment, gambling, or insurance domain using a drop-down field with the three possible options. Responses per task are reported in Fig. 4. The means of the preference stability index for the six pairwise comparisons of risk preference elicitation methods, separated by attributions to the same domain or different domains, are reported in Tab. 4.

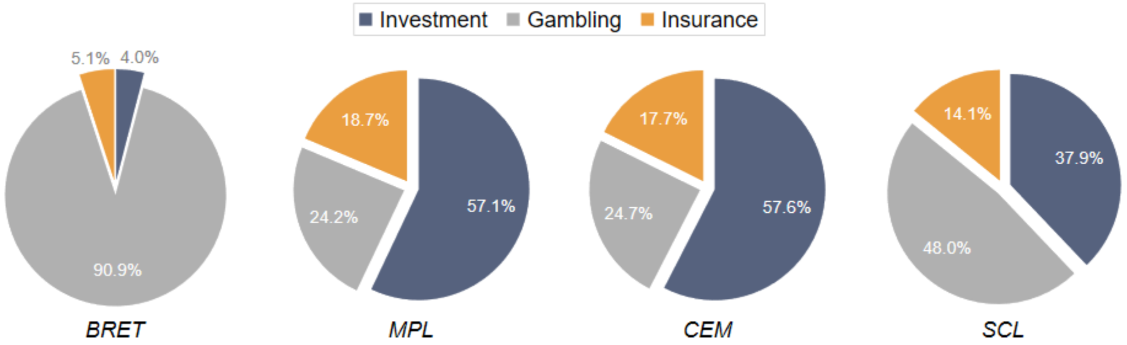

Figure 4: Subjects' attribution of domains—(i) investment, (ii) gambling, and (iii) insurance—separated by risk preference elicitation methods. $n = 198$. BRET, CEM, MPL, and SCL denote the "bomb" risk elicitation task, the certainty equivalent method, the multiple price list, and the single choice list, respectively.

Table 4: Means of the preference stability index for the six pairwise comparisons of risk preference elicitation methods, separated by whether the tasks are perceived to belong to the same domain or to different domains. Number of observations per class are reported in parentheses. Test statistics and p-values of $\chi^2(1)$-tests on differences between "same domain" and "different domain" are depicted in the lower panel.

Domain …	BRET−CEM	BRET−MPL	BRET−SCL	CEM−MPL	CEM−SCL	MPL−SCL
same	50.0% (42)	21.7% (46)	35.8% (81)	54.2% (118)	70.8% (89)	50.0% (86)
different	50.4% (143)	20.9% (139)	33.7% (104)	68.7% (67)	68.8% (96)	48.5% (99)
$\chi^2(1)$	0.002	0.016	0.093	3.686	0.091	0.042
p-value	0.968	0.900	0.761	0.055	0.763	0.837

Appendix 4. Preference Stability Index

Experimental data vs. simulation outcomes. Fig. 1 in the main text in the main text shows the distribution of the preference stability index observed in the experiment as well as the results from three different simulation exercises. The distributions depicted in Panels B and D clearly differ from the distribution of stability index resulting from the experimental data in location and shape. However, the question how to properly compare the distributions depicted in Panels A and C of Fig. 1—which seem to share many similarities—by means of a statistical test requires some consideration. In the simulation exercise, choices for each of the four tasks are drawn independently from the choice distribution observed in the experiment. To avoid sampling errors, we choose a sample size of $n = 10,000$ in all simulation exercises—a number that is sufficiently large to ensure that the simulated distribution resembles the distribution of choices per task observed in the experiment. However, comparing the sample of $n = 185$ in the experiment with the $n = 10,000$ simulated subjects using a Kolmogorov-Smirnov test is not a sensible means to examine whether the distributions differ in location and/or shape, since the test's critical value is inversely related to the number of observations.

We thus re-run the simulation exercise with $n = 185$ observations. To avoid potential sampling biases, we repeat this exercise 10,000 times, with randomly varied seeds—i.e., we apply a Bootstrapping procedure. The results of this supplementary analysis are not conclusive: The Kolmogorov-Smirnov statistic $D = \sup_x |F_{1,n}(x) - F_{2,m}(x)|$ varies from 0.049 to 0.190 and suggests that the difference between the distributions is statistically insignificant in about 70% of the iterations; the remaining 30% of iterations yield p-values below the 5% significance threshold.

Since p-values are uniformly distributed under the null hypothesis, we would expect a share of 5% significant results (false positives) if there would in fact be no difference between the distributions (i.e., if the null hypothesis would be actually true). Thus, our finding that about 30% of the iteration runs result in statistically significant differences could be an indication that the alternative hypothesis is true, i.e., that the distributions do indeed differ, but the test is not sufficiently powered to reliably detect the effect. Assuming that the alternative hypothesis is true, the comparison of distributions with $n_1 = n_2 = 185$ would only have a statistical power of about 30%, suggesting that the true effect size might be rather small.

Preference Stability per Task. Apparently, the four risk preference elicitation methods differ substantially in the number of choices, the mapping of choices into CRRA parameter intervals, and the range of the codomain. Given the distinct mechanics of the four tasks, the "contribution" of each task to the overall preference stability index may vary. Our conclusions about the extent of preference (in)stability might thus be driven by only one or two tasks.

Splitting up the overall index into its components (i.e., the six pairwise comparisons) reveals substantial variation: the share of subjects with overlapping CRRA intervals vary between 21.1% and 68.1%. All pairwise differences between the pairwise stability scores are statistically significant at a 5% level (Wilcoxon signed rank tests), except for the differences between (i) BRET–CEM and MPL–CEM and (ii) CEM–MPL and CEM–SCL. Given the small number of tasks, however, it is not possible to reliably infer whether this variation is attributable to particular mechanics of particular methods.

To examine whether any of the tasks stands out in terms of its contribution to the overall preference stability index, we determine the preference stability index on a per-task basis. In particular, we construct an index for each of the four risk preference elicitation methods separately, restricting our attention to those pairwise comparisons of methods in which the particular task is involved. For each task, we construct a measure aggregating the binary indicators of whether the CRRA parameter interval associated with the choice in a particular task overlap with the CRRA parameter intervals of the three other tasks

in the experiment. That is, the index for the BRET, for instance, captures the comparisons BRET–CEM, BRET–MPL, and BRET–SCL.[2]

The distributions of indices per task – together with the distribution of scores resulting from a simulation exercise assuming independent draws from the empirical distributions – are presented in Fig. 5. Apparently, the distributions of the preference stability indices associated with each task vary in both shape and location. While the median score is 1 out of 3 for the BRET and the MPL, it is 2 out of 3 for the CEM and the SCL. The means vary between 1.06 (BRET) and 1.78 (CEM), with standard deviations varying between 0.82 (MPL) and 1.09 (CEM). This variation might stem from a number of task-specific mechanics such as the number of choices (e.g., the size of the implied parameter intervals), the mapping from choices into CRRA parameter intervals (e.g., the symmetry of interval ranges), or the range of the codomain (e.g., the availability of risk-loving preferences). Yet, even though the contribution of the four tasks does not appear to be unitary, the patterns of per-task preference stability indicate that there is substantial variation in revealed preferences across methods. Put differently, the per-task analysis suggests that the results based on the aggregate measure (as reported in the main text) seem to be robust, and that all of the tasks contribute to the overall preference stability index.

It is reassuring that the overall indication of the simulation exercise assuming independence between the tasks (see Figure 5) appear robust on the per-task level too. Similar as for the aggregate index, the preference stability index in the simulations on a per-task basis turns out to be slightly smaller than the indices observed in the experimental data; yet, again, the shape and location of the distributions resulting from the simulations per task share considerable similarities with the experimental data. In order to test whether the preference stability indices in the experiment differ from the simulation exercises, we replicate the Bootstrapping approach – as described above – per task, i.e., we conduct 10,000 simulation runs with $n = 185$ and compare the empirical distribution and the simulation results using Kolmogorov-Smirnov tests. For the BRET, zero out of 10,000 iterations are statistically significant ($p < 0.05$); for the CEM and MPL, 14.77% and 10.24% of the simulations are significantly different from the empirical distribution, respectively; for the SCL, however, the distributions significantly differ in 9,091 of 10,000 runs. These findings – particularly with respect to the BRET and the SCL – might be indicative of a systematic effect of the size of parameter intervals and/or the range of the codomain on our aggregate measure of preference stability. However, since our setting does not involve a systematic variation of these potential determinants, we abstain from overemphasizing these patterns. We leave the question of whether and how mechanics of different tasks translate into (different measures of) preference stability to future research. However, our main finding on the risk elicitation puzzle, i.e., that there is ample heterogeneity of individual risk preferences across methods that needs to be explained, resonates in the per-task perspective.

[2] The way the index is constructed implies that this analysis considers each of the six pairwise comparisons twice; e.g., the comparison BRET–CEMis part of both the index associated with the BRET and the index associated with the CEM. This is why the distributions of the preference stability indices of two tasks cannot be reasonably compared using statistical tests.

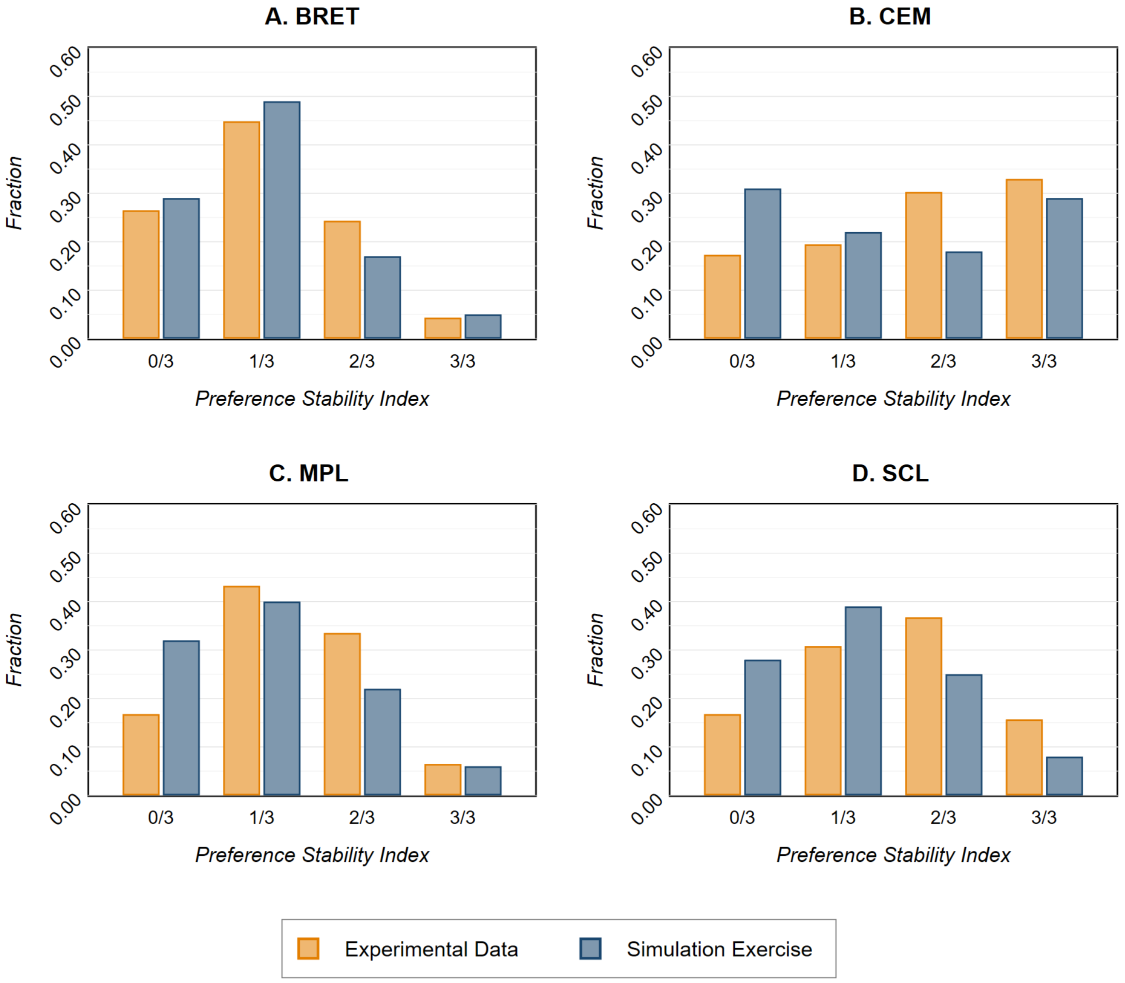

Figure 5: Distributions of the per-task preference stability index based on the experimental data (orange bars; $n = 185$) and based on simulation exercises assuming 10,000 virtual subjects choosing independently from the choice distribution of each task observed in the experiment . BRET, CEM, MPL, and SCL denote the "bomb" risk elicitation task, the certainty equivalent method, the multiple price list, and the single choice list, respectively.

Appendix 5. Order Effects

To prevent that the ordering of risk preference elicitation procedures systematically affects subjects' risk-taking behavior, the sequence of tasks in the experiment was randomized at the subject level. Yet, despite the randomization, one could hypothesize that risk preferences, and/or subjects' susceptibility to making mistakes in evaluating the alternatives, might be affected by the task ordering. For instance, it might be the case that subjects try to balance the overall risk they take in the experiment, in which case subjects would take systematically more (less) risk if their decision in the previous task involves a low (high) level of risk. Likewise, due to learning effects, subjects might be less prone to making errors in evaluating the choices in tasks that appear towards the end of the sequence; or, on the contrary, one could argue that fatigue increases the likelihood of making mistakes.

To rule out that spurious effects drive the results reported in the main text, Tab. 5 and 6 summarize additional analyses examining potential order effects. In particular, Panel A in Tab. 5 reports maximum likelihood estimates of the structural model as described in Section 4 in the main text for each of the four positions in the random task sequence; Panel B in Tab. 5 shows the pairwise differences in point estimates of φ and σ between the four positions in the sequence. Apparently, none of the differences—neither for the CRRA coefficient φ nor for the standard deviation of the noise parameter σ—is statistically significantly different from zero.

Table 5: (A) Maximum likelihood estimates of structural models with Fechner error terms for each of the four positions in the random task sequence. Standard errors, clustered on the subject level, are reported in parentheses. **(B)** Pairwise differences in point estimates of risk preference parameters φ (lower-triangular matrix) and the standard deviation of noise parameters σ (upper-triangular matrix) between the four positions in the task sequence. p-values are based on pairwise Wald tests. * $p < 0.05$, ** $p < 0.01$, *** $p < 0.001$.

Panel A	Order = 1	Order = 2	Order = 3	Order = 4
φ	0.601***	0.645***	0.541***	0.548***
	(0.057)	(0.059)	(0.054)	(0.050)
σ	0.346***	0.253***	0.365***	0.348***
	(0.064)	(0.046)	(0.072)	(0.066)
$\ln L$	−2,753	−4,222	−2,804	−3,250
No. of Obs.	5,306	7,798	5,346	6,102
Clusters	198	198	198	198

Panel B	Order = 1	Order = 2	Order = 3	Order = 4
Order = 1		0.093	−0.019	−0.001
Order = 2	−0.044		−0.112	−0.094
Order = 3	0.060	0.104		0.018
Order = 4	0.053	0.097	−0.007	

To rule out that our main findings are impaired by some systematic effect of a particular task on its succeeding one, we estimate the structural model for each of the four risk preference elicitation methods, controlling for the preceding one in the random sequence. Indeed, as depicted in Tab. 6, none of the dichotomous controls—neither for φ nor σ—turns out to be statistically significant, suggesting that our results are not affected by potential interrelations between preceding and succeeding tasks in the ordering.

Table 6: Maximum likelihood estimates of structural models with Fechner error terms for each of the four risk preference elicitation methods, controlling for the preceding task. BRET, CEM, MPL, and SCL denote the "bomb" risk elicitation task, the certainty equivalent method, the multiple price list, and the single choice list, respectively. * $p < 0.05$, ** $p < 0.01$, *** $p < 0.001$.

	BRET	CEM	MPL	SCL
φ (Constant)	0.680***	0.967***	0.615***	0.321***
	(0.042)	(0.133)	(0.070)	(0.071)
Prev. Task = BRET		−0.283	0.013	0.077
		(0.226)	(0.093)	(0.093)
Prev. Task = CEM	−0.036		−0.033	0.058
	(0.056)		(0.090)	(0.095)
Prev. Task = MPL	−0.078	−0.333		0.128
	(0.061)	(0.237)		(0.104)
Prev. Task = SCL	−0.111	0.006	−0.028	
	(0.060)	(0.273)	(0.101)	
σ (Constant)	0.044***	0.184***	0.954***	0.707***
	(0.004)	(0.051)	(0.126)	(0.110)
Prev. Task = BRET		0.151	−0.087	−0.065
		(0.127)	(0.160)	(0.150)
Prev. Task = CEM	0.005		−0.172	0.030
	(0.007)		(0.177)	(0.153)
Prev. Task = MPL	−0.002	0.218		0.095
	(0.005)	(0.168)		(0.182)
Prev. Task = SCL	−0.001	0.048	0.243	
	(0.006)	(0.125)	(0.201)	
$\ln L$	−5,235	−452	−592	−571
No. of Obs.	19,800	1,782	1,980	990
Clusters	198	198	198	198

The analyses above only provide insights into potential effects of the task ordering on the estimates of the mean parameters in the structural model, but not on individual-level estimates of the stability of preferences. To address the latter, we examine potential order effects with respect to the preference stability index defined in Section 4 in the main text. Given that there are four tasks, each of which might take any of the four positions in the sequence, there are $n = 4!$ possible permutations.[3] To

[3] For the randomization in our experiment it turns out that each of the 24 possible permutations was realized for at least two subjects, whereas the maximum number of subjects who faced the same permutation was 19.

evaluate whether specific permutations induce significantly different stability indices, we conduct 24 t-tests, each comparing the mean stability index of one particular permutation to the mean stability index of the remaining 23 task sequences. It turns out that the stability indices do not statistically differ from other permutations for any of the 24 different sequences (t-values vary between 0.033 and 1.754; corresponding p-value range from 0.973 to 0.081, respectively). Overall, the analyses summarized above provide strong evidence that the results presented in the main text are not spurious in the sense that they might be the result of order or learning effects.

Appendix 6. Random Parameter Model

Apesteguia and Ballester (2018) prove that random utility models may violate monotonicity in the sense that the probability of choosing the more risky alternative in a binary choice setting is an increasing function of the risk preference parameter. Yet, Apesteguia and Ballester (2018) show that random parameter models, as introduced by Eliashberg and Hauser (1985) and Loomes and Sugden (1995), are always monotone in the choice probability.

In contrast to binary random utility models, where the noise term is modelled to distort the evaluation of expected utilities of the two alternatives, the noise term in random parameter models distorts the decision maker's preference parameter. That is, the decision maker is assumed to choose the alternative that maximizes the utility given a particular coefficient of risk aversion φ, distorted by a common random error ϵ. In the random parameter model, the probability of choosing alternative B has the closed form of $e^{\lambda\varphi^*}/(e^{\lambda\varphi^*} + e^{\lambda\varphi})$, where φ^* refers to the CRRA parameter which equates the expected utilities of the two lotteries, i.e., $u_{\varphi^*}(A) = u_{\varphi^*}(B)$, and λ denotes a precision parameter which is inversely related to the variance of a random noise term (Apesteguia and Ballester, 2018). Note that a decrease in φ (i.e., a decrease in risk aversion) implies a decrease in the denominator, guaranteeing that the choice probability increases and monotonicity is preserved.

In addition to the estimates based on the random utility model reported in the main text, we therefore report estimates of the random parameter model in Tab. 7. It is reassuring that our main findings are corroborated by qualitatively similar results: While the point estimates of the CRRA parameter φ turn out to be higher for all tasks (which is in line with the results reported by Apesteguia and Ballester, 2018), the ordering of parameter estimates across tasks is preserved and the patterns of statistically significant differences between tasks remain similar.

Despite the advantage of the random parameter model, we chose to report results in the Appendix rather than the main text for two reasons: First, the value of φ^* is practically not determinable for the dominated choices in the BRET and CEM, i.e., for 2 out of 9 binary choices in the CEM and for 2 out of 99 binary choices in the BRET. Applying the random parameter model, thus, implies that 396 observations need to be dropped from the analysis for both the BRET and the CEM. Second, the solutions for the values of φ^* turn out to be labile for several binary choices, in particular for very low ($k < 5$) and very high ($k > 90$) numbers of selected boxes in the BRET, but also for the most and least risk averse decisions in the other three tasks. Given the properties of the power utility function, solutions to the non-linear equations are overly sensitive to marginal deviations and, thus, computationally hard to approximate. The extent to which the parameter estimates of a particular task are impaired by these effects may well be systematic in nature since the parameter ranges covered by the different elicitation procedures—and thereby the values of the solutions for φ^* and, as a result, the precision of solutions for φ^* — vary substantially. Irrespective of which model is considered superior given our data, it is eventually reassuring that our main results are robust in both specifications.

Table 7: (A) Maximum likelihood estimates of random parameter models for each of the four risk preference elicitation methods. Standard errors, clustered on the subject level, are reported in parentheses. **(B)** Pairwise differences in point estimates of risk preference parameters φ (lower-triangular matrix) and precision parameters λ (upper-triangular matrix) between the four risk preference elicitation methods. p-values are based on pairwise Wald tests. BRET, CEM, MPL, and SCL denote the "bomb" risk elicitation task, the certainty equivalent method, the multiple price list, and the single choice list, respectively. * $p < 0.05$, ** $p < 0.01$, *** $p < 0.001$.

Panel A	BRET	CEM	MPL	SCL
φ	0.653***	0.896***	0.696***	0.579***
	(0.023)	(0.004)	(0.012)	(0.014)
$ln\lambda$	3.733***	2.999***	4.056***	4.837***
	(0.174)	(0.851)	(0.052)	(0.650)
$\ln L$	−12,153	−838	−1,207	−536
No. of Obs.	19,404	1,386	1,980	990
Clusters	198	198	198	198

Panel B	BRET	CEM	MPL	SCL
BRET		0.779***	−0.320	−0.947***
CEM	0.241***		−1.099***	−1.726***
MPL	0.043	−0.199***		−0.627*
SCL	−0.078*	−0.320***	−0.121*	

Appendix 7. Screenshots of the Experiment

In the following, we present annotated screenshots of each page of the experiment, containing all instructions (translated to English). Each page corresponds to a single screen as displayed to subjects in the experiment.

Screen 1: *Welcome*

Note: *The experimental sessions took place at the Innsbruck EconLab and the were conducted in German. The following screenshots depict all screens of the experiment translated to English.*

Innsbruck EconLab

Dear participants,

Welcome to today's experiment.

Please read the following instructions carefully. All statements in the instructions are true. The payments at the end of the experiment are dependent on how well you understand the instructions. If you have a question, please raise your hand. Your question will then be answered in private. The experiment and the data analysis are conducted anonymously.

From now on, please do not talk to the other participants and only use tools that are explicitly provided by the experimenters. Please also turn off any electronic devices. You may only use programs on your computer that are explicitly intended for the experiment. If you violate any of these rules you will not be paid out in this experiment and excluded from any future experiments.

Thank you very much for your attention and for attending today's experiment.

Screen 2: Overview

General Introduction

Note: *All experimental instructions were displayed on screen.*

This experiment consists of **three blocks**. Instructions for each of the blocks will be provided separately before a new block starts.

For participating in today's experiment, you will earn a **show-up fee of €4.00**. In the course of the experiment, you can earn additional money. You will only be informed about your final payment at the end of the experiment.

Screen 3: *Instructions – Block 1*

Note: *To allow for reference, the four tasks were labeled with color names. The order of tasks was randomized.*

Block 1

Block 1 consists of **four different tasks** in which you can earn money:
Task "orange", Task "blue", Task "green", and Task "red".

Potential payments are calculated separately for each of the tasks. At the end of the experiment, one of the four tasks is drawn randomly and paid out. However, you will only be informed about which of the tasks has been drawn and how much money you earn at the end of the experiment.

Screen 4: Single Choice List (SCL) – Instructions

Task 'orange': Instructions

In Task 'orange' you face six different lotteries. Each lottery has two possible outcomes (Event A or Event B). Either of the two events occurs with a 50% probability.

Your task is to indicate which of the six lotteries you would like to play. For the lottery that you choose, a random draw at the end of the experiment determines which event, Event A or Event B, occurs. This randomly drawn event determines your payment for Task 'orange'.

Screen 5: Single Choice List (SCL) – Risk Preference Elicitation

Task 'orange': Your Decision

	Event A		Event B		
**No.**	**Prob.**	**Payoff**	**Prob.**	**Payoff**	**Your Choice**
1	50.0%	€9.00	50.0%	€9.00	○
2	50.0%	€7.50	50.0%	€12.00	○
3	50.0%	€6.00	50.0%	€15.00	○
4	50.0%	€4.50	50.0%	€18.00	○
5	50.0%	€3.00	50.0%	€21.00	○
6	50.0%	€0.00	50.0%	€24.00	○

Task 'orange': Your Decision

	Event A		Event B		
**No.**	**Prob.**	**Payoff**	**Prob.**	**Payoff**	**Your Choice**
1	50.0%	€9.00	50.0%	€9.00	○
2	50.0%	€7.50	50.0%	€12.00	○
3	50.0%	€6.00	50.0%	€15.00	○
4	50.0%	€4.50	50.0%	€18.00	◉
5	50.0%	€3.00	50.0%	€21.00	○
6	50.0%	€0.00	50.0%	€24.00	○

Please answer the following questions:

On a scale from 1 (not at all risky) to 7 (very risky):
How risky do you consider your own decision (indicated above) to be?

1	*2*	*3*	*4*	*5*	*6*	*7*
○	○	○	○	○	○	○
not risky at all						*very risky*

On a scale from 1 (not at all confident) to 7 (very confident):
How confident do you feel with your decision indicated above?

1	*2*	*3*	*4*	*5*	*6*	*7*
○	○	○	○	○	○	○
not confident at all						*very confident*

Task 'blue': Instructions

In Task 'blue' you face ten rows representing ten decisions you have to make. Each row is a paired choice between Option A and Option B. You have to make ten choices, one for each row, between Option A and Option B. The possible payoffs of Option A and Option B are the same for all ten rows. Only the corresponding probabilities to earn the high and low payoff, respectively, differ in for each decision. However, only one of them determines your earnings in the end of Task 'blue'.

After you have made all of your choices, two random draws determine your payoff:

- The first draws one of the ten rows with equal probability.
- For this randomly drawn row, the second random draw determines whether the high or low lottery outcome is paid, depending on the respective probabilities for the lotteries in Option A and Option B.

Screen 8: Multiple Price List (MPL) – Risk Preference Elicitation

Task 'blue': Your Decision

Option A	Option B
€10.00 with a probability of **10.00%**, €8.00 otherwise	€19.25 with a probability of **10.00%**, €0.50 otherwise
€10.00 with a probability of **20.00%**, €8.00 otherwise	€19.25 with a probability of **20.00%**, €0.50 otherwise
€10.00 with a probability of **30.00%**, €8.00 otherwise	€19.25 with a probability of **30.00%**, €0.50 otherwise
€10.00 with a probability of **40.00%**, €8.00 otherwise	€19.25 with a probability of **40.00%**, €0.50 otherwise
€10.00 with a probability of **50.00%**, €8.00 otherwise	€19.25 with a probability of **50.00%**, €0.50 otherwise
€10.00 with a probability of **60.00%**, €8.00 otherwise	€19.25 with a probability of **60.00%**, €0.50 otherwise
€10.00 with a probability of **70.00%**, €8.00 otherwise	€19.25 with a probability of **70.00%**, €0.50 otherwise
€10.00 with a probability of **80.00%**, €8.00 otherwise	€19.25 with a probability of **80.00%**, €0.50 otherwise
€10.00 with a probability of **90.00%**, €8.00 otherwise	€19.25 with a probability of **90.00%**, €0.50 otherwise
€10.00 with a probability of **100.00%**, €8.00 otherwise	€19.25 with a probability of **100.00%**, €0.50 otherwise

Screen 9: *Multiple Price List (MPL) – Riskiness & Confidence*

Task 'blue': Your Decision

Option A			Option B
€10.00 with a probability of **10.00%**, €8.00 otherwise	●	○	€19.25 with a probability of **10.00%**, €0.50 otherwise
€10.00 with a probability of **20.00%**, €8.00 otherwise	●	○	€19.25 with a probability of **20.00%**, €0.50 otherwise
€10.00 with a probability of **30.00%**, €8.00 otherwise	●	○	€19.25 with a probability of **30.00%**, €0.50 otherwise
€10.00 with a probability of **40.00%**, €8.00 otherwise	●	○	€19.25 with a probability of **40.00%**, €0.50 otherwise
€10.00 with a probability of **50.00%**, €8.00 otherwise	●	○	€19.25 with a probability of **50.00%**, €0.50 otherwise
€10.00 with a probability of **60.00%**, €8.00 otherwise	●	○	€19.25 with a probability of **60.00%**, €0.50 otherwise
€10.00 with a probability of **70.00%**, €8.00 otherwise	○	●	€19.25 with a probability of **70.00%**, €0.50 otherwise
€10.00 with a probability of **80.00%**, €8.00 otherwise	○	●	€19.25 with a probability of **80.00%**, €0.50 otherwise
€10.00 with a probability of **90.00%**, €8.00 otherwise	○	●	€19.25 with a probability of **90.00%**, €0.50 otherwise
€10.00 with a probability of **100.00%**, €8.00 otherwise	○	●	€19.25 with a probability of **100.00%**, €0.50 otherwise

Please answer the following questions:

On a scale from 1 (not at all risky) to 7 (very risky):
How risky do you consider your own decision (indicated above) to be?

1	*2*	*3*	*4*	*5*	*6*	*7*
○	○	○	○	○	○	○
not risky at all						*very risky*

On a scale from 1 (not at all confident) to 7 (very confident):
How confident do you feel with your decision indicated above?

1	*2*	*3*	*4*	*5*	*6*	*7*
○	○	○	○	○	○	○
not confident at all						*very confident*

Task 'green': Instructions

In Task 'green' you face nine rows representing nine decisions you have to make. Each row is a paired choice between Option A and Option B. Option A is a lottery paying €15.00 in 50.0% of the cases and €5.00 otherwise. The lottery is the same in each of the nine rows. Option B is a sure payoff, varying in each of the nine rows. You have to make nine choices, one for each row, between Option A and Option B. However, only one of them determines your earnings in the end of Task 'green'.

After you have made all of your choices, up to two random draws determine your payoff:

- The first draws one of the nine rows with equal probability.
- If you have chosen Option A for this randomly drawn row, a second random drawn determines your payment which is either €15.00 or €5.00 with equal probabilities.
- If you have chosen Option B for this randomly drawn row, you earn the respective sure payoff.

Screen 11: Certainty Equivalent Method (CEM) – Risk Preference Elicitation

Task 'green': Your Decision

Option A			Option B
€15.00 with a probability of 50%, €5.00 otherwise	○	○	**€5.00** with a probability of 100% (sure payoff)
€15.00 with a probability of 50%, €5.00 otherwise	○	○	**€6.25** with a probability of 100% (sure payoff)
€15.00 with a probability of 50%, €5.00 otherwise	○	○	**€7.50** with a probability of 100% (sure payoff)
€15.00 with a probability of 50%, €5.00 otherwise	○	○	**€8.75** with a probability of 100% (sure payoff)
€15.00 with a probability of 50%, €5.00 otherwise	○	○	**€10.00** with a probability of 100% (sure payoff)
€15.00 with a probability of 50%, €5.00 otherwise	○	○	**€11.25** with a probability of 100% (sure payoff)
€15.00 with a probability of 50%, €5.00 otherwise	○	○	**€12.50** with a probability of 100% (sure payoff)
€15.00 with a probability of 50%, €5.00 otherwise	○	○	**€13.75** with a probability of 100% (sure payoff)
€15.00 with a probability of 50%, €5.00 otherwise	○	○	**€15.00** with a probability of 100% (sure payoff)

Task 'green': Your Decision

Option A			Option B
€15.00 with a probability of 50%, €5.00 otherwise	◉	○	**€5.00** with a probability of 100% (sure payoff)
€15.00 with a probability of 50%, €5.00 otherwise	◉	○	**€6.25** with a probability of 100% (sure payoff)
€15.00 with a probability of 50%, €5.00 otherwise	◉	○	**€7.50** with a probability of 100% (sure payoff)
€15.00 with a probability of 50%, €5.00 otherwise	◉	○	**€8.75** with a probability of 100% (sure payoff)
€15.00 with a probability of 50%, €5.00 otherwise	○	◉	**€10.00** with a probability of 100% (sure payoff)
€15.00 with a probability of 50%, €5.00 otherwise	○	◉	**€11.25** with a probability of 100% (sure payoff)
€15.00 with a probability of 50%, €5.00 otherwise	○	◉	**€12.50** with a probability of 100% (sure payoff)
€15.00 with a probability of 50%, €5.00 otherwise	○	◉	**€13.75** with a probability of 100% (sure payoff)
€15.00 with a probability of 50%, €5.00 otherwise	○	◉	**€15.00** with a probability of 100% (sure payoff)

Please answer the following questions:

On a scale from 1 (not at all risky) to 7 (very risky):
How risky do you consider your own decision (indicated above) to be?

1	2	3	4	5	6	7
○	○	○	○	○	○	○
not risky at all						*very risky*

On a scale from 1 (not at all confident) to 7 (very confident):
How confident do you feel with your decision indicated above?

1	2	3	4	5	6	7
○	○	○	○	○	○	○
not confident at all						*very confident*

Task 'red': Instructions

In Task 'red' you face a square composed of 100 boxes. You can earn €0.50 for each box that you collect. However, a bomb is hidden in one of the boxes, but you do not know in which one. You only know that the bomb can be located in any box with equal probability.

As soon as you start the task by clicking on the "Start" button, one of the boxes is collected every second. Collected boxes are marked by a tick symbol. Your task is to choose when to stop the collection process by hitting the "Stop" button. Once you have clicked on the "Stop" button you cannot continue collecting boxes.

If the bomb is among the collected boxes, it destroys everything and your payoff is zero. If the bomb in not among the boxes that you have collected, your payoff is €0.50 for each box collected.

Screen 14: *Bomb Risk Elicitation Task (BRET) – Risk Preference Elicitation*

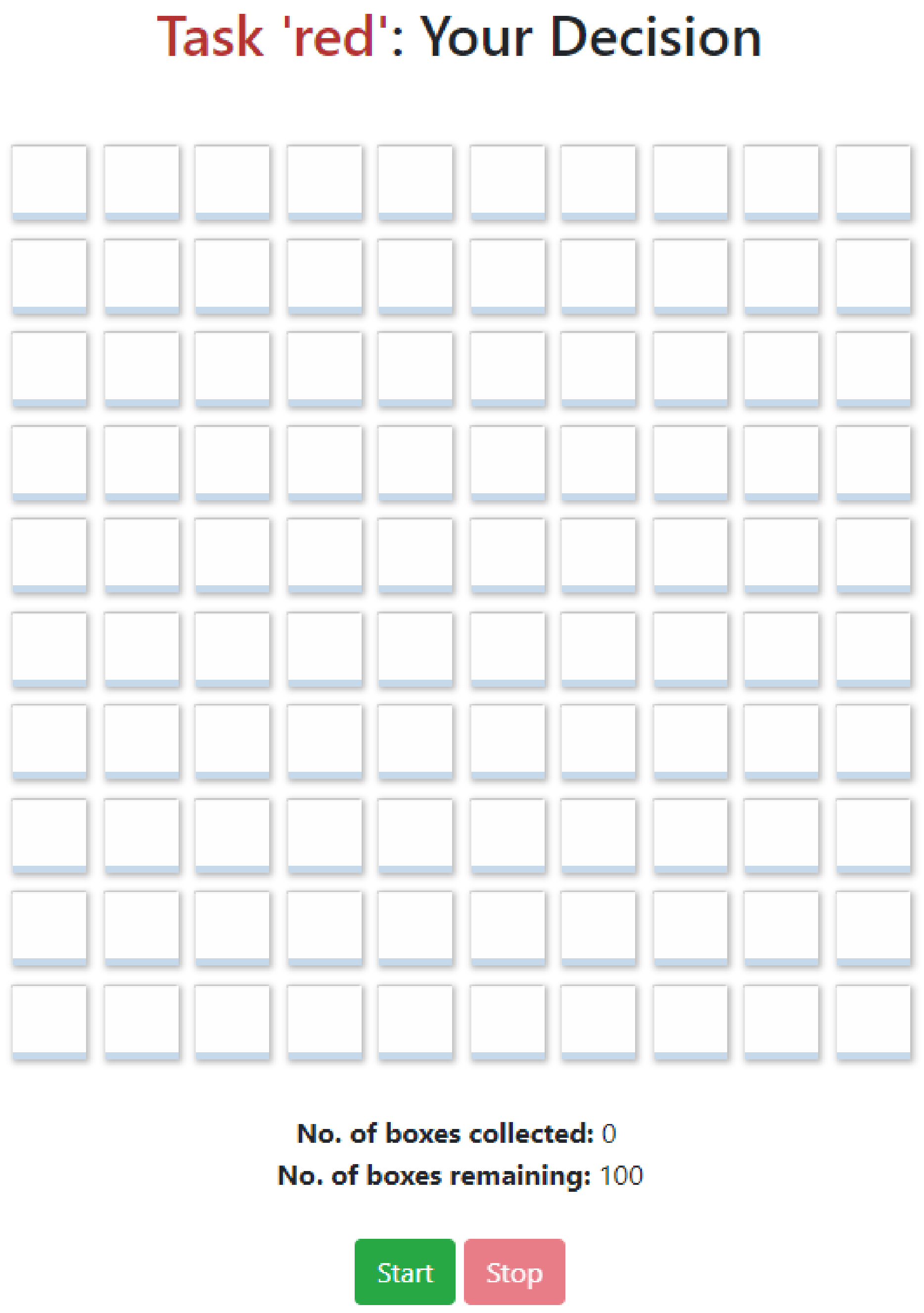

Screen 15: *Bomb Risk Elicitation Task (BRET) – Riskiness & Confidence*

Task 'red': Your Decision

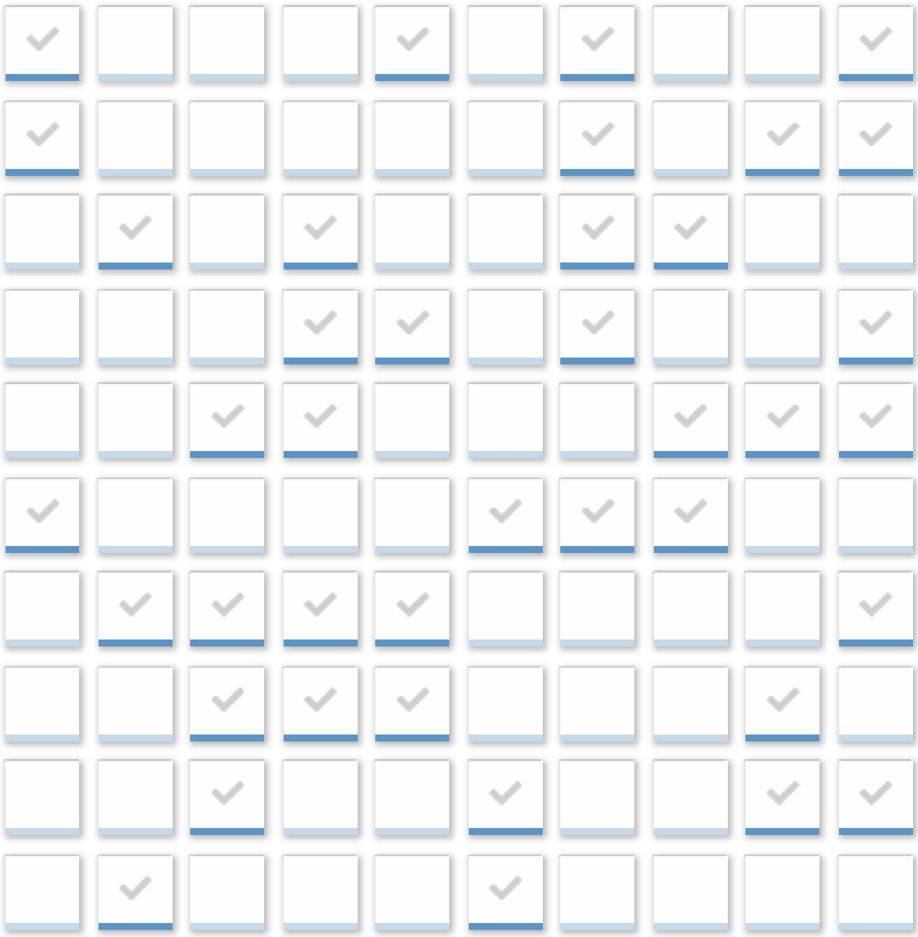

No. of boxes collected: 40
No. of boxes remaining: 60

Please answer the following questions:

On a scale from 1 (not at all risky) to 7 (very risky):
How risky do you consider your own decision (indicated above) to be?

1	2	3	4	5	6	7
○	○	○	○	○	○	○

not risky at all … *very risky*

On a scale from 1 (not at all confident) to 7 (very confident):
How confident do you feel with your decision indicated above?

1	2	3	4	5	6	7
○	○	○	○	○	○	○

not confident at all … *very confident*

Screen 16: Instructions – Block 2

Note: *To avoid confusion, the order of tasks remained the same throughout the experiment.*

Block 2

In Block 2, you are asked to compare the decisions in Task "orange" , Task "blue" , Task "green" , and Task "red" of Block 1 and to answer questions on particular decisions made in each task.

To do so, you are first shortly reminded of the four tasks by reviewing sample screenshots of the decision problems and a summary of the respective instructions.

At any point you can read the instructions of the four tasks by clicking on the respective symbol.

For carefully and truthfully answering the questions in Block 2, you receive **€3.00**.

Screen 17: Reminder – Single Choice List (SCL)

Task 'orange'

In Task 'orange' you face six different lotteries. Each lottery has two possible outcomes (Event A or Event B). Either of the two events occurs with a 50% probability.

Your task is to indicate which of the six lotteries you would like to play. For the lottery that you choose, a random draw at the end of the experiment determines which event, Event A or Event B, occurs. This randomly drawn event determines your payment for Task 'orange'.

	Ereignis A		Ereignis B		
**Nr.**	Wahrsch.	**Auszahlung**	Wahrsch.	**Auszahlung**	**Ihre Wahl**
1	50.0%	9,00 €	50.0%	9,00 €	○
2	50.0%	7,50 €	50.0%	12,00 €	○
3	50.0%	6,00 €	50.0%	15,00 €	○
4	50.0%	4,50 €	50.0%	18,00 €	○
5	50.0%	3,00 €	50.0%	21,00 €	○
6	50.0%	0,00 €	50.0%	24,00 €	○

Screen 18: *Reminder – Multiple Price List (MPL)*

Task 'blue'

In Task 'blue' you face ten rows representing ten decisions you have to make. Each row is a paired choice between Option A and Option B. You have to make ten choices, one for each row, between Option A and Option B. The possible payoffs of Option A and Option B are the same for all ten rows. Only the corresponding probabilities to earn the high and low payoff, respectively, differ in for each decision. However, only one of them determines your earnings in the end of Task 'blue'.

Alternative A	Alternative B
10,00 € mit einer Wahrscheinlichkeit von **10.00%**, 8,00 € anderenfalls	19,25 € mit einer Wahrscheinlichkeit von **10.00%**, 0,50 € anderenfalls
10,00 € mit einer Wahrscheinlichkeit von **20.00%**, 8,00 € anderenfalls	19,25 € mit einer Wahrscheinlichkeit von **20.00%**, 0,50 € anderenfalls
10,00 € mit einer Wahrscheinlichkeit von **30.00%**, 8,00 € anderenfalls	19,25 € mit einer Wahrscheinlichkeit von **30.00%**, 0,50 € anderenfalls
10,00 € mit einer Wahrscheinlichkeit von **40.00%**, 8,00 € anderenfalls	19,25 € mit einer Wahrscheinlichkeit von **40.00%**, 0,50 € anderenfalls
10,00 € mit einer Wahrscheinlichkeit von **50.00%**, 8,00 € anderenfalls	19,25 € mit einer Wahrscheinlichkeit von **50.00%**, 0,50 € anderenfalls
10,00 € mit einer Wahrscheinlichkeit von **60.00%**, 8,00 € anderenfalls	19,25 € mit einer Wahrscheinlichkeit von **60.00%**, 0,50 € anderenfalls
10,00 € mit einer Wahrscheinlichkeit von **70.00%**, 8,00 € anderenfalls	19,25 € mit einer Wahrscheinlichkeit von **70.00%**, 0,50 € anderenfalls
10,00 € mit einer Wahrscheinlichkeit von **80.00%**, 8,00 € anderenfalls	19,25 € mit einer Wahrscheinlichkeit von **80.00%**, 0,50 € anderenfalls
10,00 € mit einer Wahrscheinlichkeit von **90.00%**, 8,00 € anderenfalls	19,25 € mit einer Wahrscheinlichkeit von **90.00%**, 0,50 € anderenfalls
10,00 € mit einer Wahrscheinlichkeit von **100.00%**, 8,00 € anderenfalls	19,25 € mit einer Wahrscheinlichkeit von **100.00%**, 0,50 € anderenfalls

Screen 19: *Reminder – Certainty Equivalent Method (CEM)*

Task 'green'

In Task 'green' you face nine rows representing nine decisions you have to make. Each row is a paired choice between Option A and Option B. Option A is a lottery paying €15.00 in 50% of the cases and €5.00 otherwise. The lottery is the same in each of the nine rows. Option B is a sure payoff, varying in each of the nine rows. You have to make nine choices, one for each row, between Option A and Option B. However, only one of them determines your earnings in the end of Task 'green'.

Alternative A			Alternative B
15,00 € mit einer Wahrscheinlichkeit von 50%, 5,00 € anderenfalls	○	○	**5,00 €** mit einer Wahrscheinlichkeit von 100% (sichere Auszahlung)
15,00 € mit einer Wahrscheinlichkeit von 50%, 5,00 € anderenfalls	○	○	**6,25 €** mit einer Wahrscheinlichkeit von 100% (sichere Auszahlung)
15,00 € mit einer Wahrscheinlichkeit von 50%, 5,00 € anderenfalls	○	○	**7,50 €** mit einer Wahrscheinlichkeit von 100% (sichere Auszahlung)
15,00 € mit einer Wahrscheinlichkeit von 50%, 5,00 € anderenfalls	○	○	**8,75 €** mit einer Wahrscheinlichkeit von 100% (sichere Auszahlung)
15,00 € mit einer Wahrscheinlichkeit von 50%, 5,00 € anderenfalls	○	○	**10,00 €** mit einer Wahrscheinlichkeit von 100% (sichere Auszahlung)
15,00 € mit einer Wahrscheinlichkeit von 50%, 5,00 € anderenfalls	○	○	**11,25 €** mit einer Wahrscheinlichkeit von 100% (sichere Auszahlung)
15,00 € mit einer Wahrscheinlichkeit von 50%, 5,00 € anderenfalls	○	○	**12,50 €** mit einer Wahrscheinlichkeit von 100% (sichere Auszahlung)
15,00 € mit einer Wahrscheinlichkeit von 50%, 5,00 € anderenfalls	○	○	**13,75 €** mit einer Wahrscheinlichkeit von 100% (sichere Auszahlung)
15,00 € mit einer Wahrscheinlichkeit von 50%, 5,00 € anderenfalls	○	○	**15,00 €** mit einer Wahrscheinlichkeit von 100% (sichere Auszahlung)

Screen 20: Reminder – 'Bomb' Risk Elicitation Task

Task 'red'

In Task 'red' you face a square composed of 100 boxes. You can earn €0.50 for each box that you collect. However, a bomb is hidden in one of the boxes, but you do not know in which one. You only know that the bomb can be located in any box with equal probability.

As soon as you start the task by clicking on the "Start" button, one of the boxes is collected every second. Collected boxes are marked by a tick symbol. Your task is to choose when to stop the collection process by hitting the "Stop" button. Once you have clicked on the "Stop" button you cannot continue collecting boxes.

Anzahl der gesammelten Boxen: 0
Anzahl der verbleibenden Boxen: 100

Start Stopp

Comparison of Tasks

Please answer the following questions for each of the four tasks.

For the first three questions, please indicate how much you agree to the respective statements using a scale from **1 (not agree at all) to 7 (fully agree)**. For the fourth question, please indicate which of the domains best describes the decision situation in the tasks.

Note: *At any time, instructions of the tasks were available using the "info" icon.*

	Task "orange"	Task "blue"	Task "green"	Task "red"
The task is easy to understand and can be answered straightforwardly.	---	---	---	---
The task involves complex calculations and requires deliberating on the trade-off between expected outcomes and the inherent riskiness of the different outcomes.	---	---	---	---
Completing the task is annoying and boring.	---	---	---	---
The task does best resemble typical decisions in the ________ domain.	---	---	---	---

Screen 22: Comprehension – Single Choice List (SCL)

Questions on Task "orange"

Please consider the following decision in Task "orange" :

	Ereignis A		Ereignis B		
**Nr.**	**Wahrsch.**	**Auszahlung**	**Wahrsch.**	**Auszahlung**	**Ihre Wahl**
1	50.0%	9,00 €	50.0%	9,00 €	○
2	50.0%	7,50 €	50.0%	12,00 €	○
3	50.0%	6,00 €	50.0%	15,00 €	○
4	50.0%	4,50 €	50.0%	18,00 €	○
5	50.0%	3,00 €	50.0%	21,00 €	◉
6	50.0%	0,00 €	50.0%	24,00 €	○

Please answer the following questions:

Please estimate how much the decision maker on average can **expect to earn** based on her decision.	[ ] Euro
Please estimate the probability that the payoff resulting from the decision indicated above are **below €5.50**.	[ ] percent
Please estimate the probability that the payoff resulting from the decision indicated above are **above €14.50**.	[ ] percent

Questions on Task "blue"

Please consider the following decision in Task "blue" :

Alternative A			Alternative B
10,00 € mit einer Wahrscheinlichkeit von **10.00%**, 8,00 € anderenfalls	●	○	19,25 € mit einer Wahrscheinlichkeit von **10.00%**, 0,50 € anderenfalls
10,00 € mit einer Wahrscheinlichkeit von **20.00%**, 8,00 € anderenfalls	●	○	19,25 € mit einer Wahrscheinlichkeit von **20.00%**, 0,50 € anderenfalls
10,00 € mit einer Wahrscheinlichkeit von **30.00%**, 8,00 € anderenfalls	●	○	19,25 € mit einer Wahrscheinlichkeit von **30.00%**, 0,50 € anderenfalls
10,00 € mit einer Wahrscheinlichkeit von **40.00%**, 8,00 € anderenfalls	●	○	19,25 € mit einer Wahrscheinlichkeit von **40.00%**, 0,50 € anderenfalls
10,00 € mit einer Wahrscheinlichkeit von **50.00%**, 8,00 € anderenfalls	○	●	19,25 € mit einer Wahrscheinlichkeit von **50.00%**, 0,50 € anderenfalls
10,00 € mit einer Wahrscheinlichkeit von **60.00%**, 8,00 € anderenfalls	○	●	19,25 € mit einer Wahrscheinlichkeit von **60.00%**, 0,50 € anderenfalls
10,00 € mit einer Wahrscheinlichkeit von **70.00%**, 8,00 € anderenfalls	○	●	19,25 € mit einer Wahrscheinlichkeit von **70.00%**, 0,50 € anderenfalls
10,00 € mit einer Wahrscheinlichkeit von **80.00%**, 8,00 € anderenfalls	○	●	19,25 € mit einer Wahrscheinlichkeit von **80.00%**, 0,50 € anderenfalls
10,00 € mit einer Wahrscheinlichkeit von **90.00%**, 8,00 € anderenfalls	○	●	19,25 € mit einer Wahrscheinlichkeit von **90.00%**, 0,50 € anderenfalls
10,00 € mit einer Wahrscheinlichkeit von **100.00%**, 8,00 € anderenfalls	○	●	19,25 € mit einer Wahrscheinlichkeit von **100.00%**, 0,50 € anderenfalls

Please answer the following questions:

Please estimate how much the decision maker on average can **expect to earn** based on her decision.	Euro
Please estimate the probability that the payoff resulting from the decision indicated above are **below €5.50**.	percent
Please estimate the probability that the payoff resulting from the decision indicated above are **above €14.50**.	percent

Screen 24: *Comprehension – Certainty Equivalent Method (CEM)*

Questions on Task "green"

Please consider the following decision in Task "green" :

Alternative A			Alternative B
15,00 € mit einer Wahrscheinlichkeit von 50%, 5,00 € anderenfalls	●	○	**5,00 €** mit einer Wahrscheinlichkeit von 100% (sichere Auszahlung)
15,00 € mit einer Wahrscheinlichkeit von 50%, 5,00 € anderenfalls	●	○	**6,25 €** mit einer Wahrscheinlichkeit von 100% (sichere Auszahlung)
15,00 € mit einer Wahrscheinlichkeit von 50%, 5,00 € anderenfalls	●	○	**7,50 €** mit einer Wahrscheinlichkeit von 100% (sichere Auszahlung)
15,00 € mit einer Wahrscheinlichkeit von 50%, 5,00 € anderenfalls	●	○	**8,75 €** mit einer Wahrscheinlichkeit von 100% (sichere Auszahlung)
15,00 € mit einer Wahrscheinlichkeit von 50%, 5,00 € anderenfalls	●	○	**10,00 €** mit einer Wahrscheinlichkeit von 100% (sichere Auszahlung)
15,00 € mit einer Wahrscheinlichkeit von 50%, 5,00 € anderenfalls	○	●	**11,25 €** mit einer Wahrscheinlichkeit von 100% (sichere Auszahlung)
15,00 € mit einer Wahrscheinlichkeit von 50%, 5,00 € anderenfalls	○	●	**12,50 €** mit einer Wahrscheinlichkeit von 100% (sichere Auszahlung)
15,00 € mit einer Wahrscheinlichkeit von 50%, 5,00 € anderenfalls	○	●	**13,75 €** mit einer Wahrscheinlichkeit von 100% (sichere Auszahlung)
15,00 € mit einer Wahrscheinlichkeit von 50%, 5,00 € anderenfalls	○	●	**15,00 €** mit einer Wahrscheinlichkeit von 100% (sichere Auszahlung)

Please answer the following questions:

Please estimate how much the decision maker on average can **expect to earn** based on her decision.	Euro
Please estimate the probability that the payoff resulting from the decision indicated above are **below €5.50**.	percent
Please estimate the probability that the payoff resulting from the decision indicated above are **above €14.50**.	percent

Screen 25: Comprehension – ‘Bomb’ Risk Elicitation Task (BRET)

Questions on Task "red"

Please consider the following decision in Task "red" :

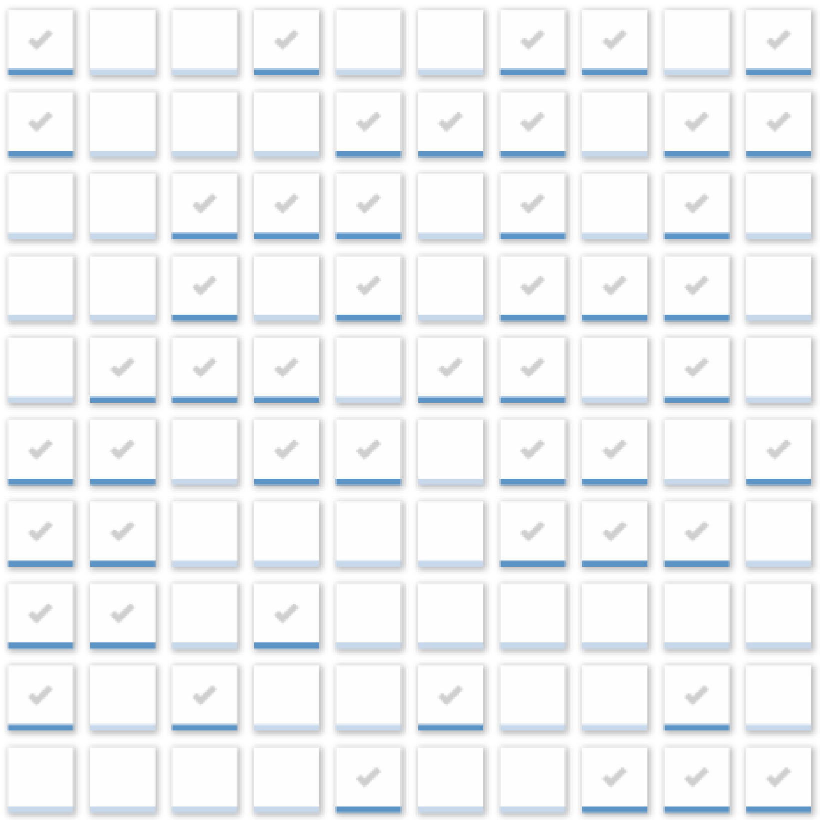

Anzahl der gesammelten Boxen: 50
Anzahl der verbleibenden Boxen: 50

Start Stopp

Please answer the following questions:

Please estimate how much the decision maker on average can **expect to earn** based on her decision.	Euro
Please estimate the probability that the payoff resulting from the decision indicated above are **below €5.50**.	percent
Please estimate the probability that the payoff resulting from the decision indicated above are **above €14.50**.	percent

Screen 26: Preferred Task

Your Preference

If you could decide ...

...which of the four tasks would you prefer to be included in future experiments?

- Task 'orange'
- Task 'blue'
- Task 'green'
- Task 'red'

Screen 27: Instructions – Block 3

Block 3

In Block 3, you have to answer eight questions. For each correct answer you receive €0.50. You only have 8 minutes for answering the questions in Block 3.

Note that you will only be informed about the number of correct answers at the end of the experiment.

Questionnaire

Time left to complete this page: **4:46**

Please answer the following question:

If John can drink one barrel of water in 6 days, and Mary can drink one barrel of water in 12 days, how long would it take them to drink one barrel of water together?	days
In the BIG BUCKS LOTTERY, the chances of winning a $10.00 prize are 1%. What is your best guess about how many people would win a $10.00 prize if 1000 people each buy a single ticket from BIG BUCKS?	people
In the ACME PUBLISHING SWEEPSTAKES, the chance of winning a car is 1 in 1000. What percent of tickets of ACME PUBLISHING SWEEPSTAKES win a car?	percent
A man buys a pig for $60, sells it for $70, buys it back for $80, and sells it finally for $90. How much has he made?	dollars
If the chance of getting a disease is 10%, how many people would be expected to get the disease out of 1000?	people
Imagine that we roll a fair, six-sided die 1000 times. Out of 1000 rolls, how many times do you think the die would come up as an even number?	times
Suppose you have a close friend who has a lump in her breast and must have a mammogram. Of 100 women like her, 10 of them actually have a malignant tumor and 90 of them do not. Of the 10 women who actually have a tumor, the mammogram indicates correctly that 9 of them have a tumor and indicates incorrectly that 1 of them does not. Of the 90 women who do not have a tumor, the mammogram indicates correctly that 81 of them do not have a tumor and indicates incorrectly that 9 of them do have a tumor. Imagine that your friend tests positive (as if she had a tumor), what is the likelihood that she actually has a tumor?	percent
If the chance of getting a disease is 20 out of 100, this would be the same as having a ...% chance of getting the disease.	percent

Screen 29: Demographics Survey

General Questions

Before concluding the experiment with your payment, please answer the following questions truthfully.

Gender:	○ Male<br>○ Female
Age:	
Have you ever invested in financial markets (stocks, bonds, portfolios, etc.)?	○ yes<br>○ no
Have you successfully completed a course in Finance, Mathematics, or Statistics at the University?	○ yes<br>○ no

Screen 30: Payment Information – Block 1

Note: *For subject's payout, one of the four tasks has been chosen randomly.*

Your Payment from Block 1

For Block 1, **Task "orange"** has been randomly drawn for your payment. On the next page, you'll be informed about the outcome of this task.

A summary of your final payment from this experiment will follow on the subsequent page.

Screen 31: *Payment Information – Block 1*

Task 'orange': Results

Note: *For the chosen task, details on the payoff-relevant decision and the random elements determining the payoff were displayed.*

You decided to choose the following lottery:

	Event A		Event B		
**No.**	**Prob.**	**Payoff**	**Prob.**	**Payoff**	**Your Choice**
4	50.0%	€4.50	50.0%	€18.00	◉

A random draw determined that event B has been realized.
Your payoff from this task is **€18.00**.

Screen 32: Final Payout Information

Your Final Payment

Thank you very much for having participated in the experiment!

Please find a summary of your final payment below.

Payoff Summary

**Show-up fee:**	€4.00
**Block 1:** (Task "orange")	€18.00
**Block 2:** (for answering all questions)	€3.00
**Block 3:** (for answering 1 of the 8 questions correctly)	€0.50

Thus, your final payoff from today's experiment is €25.50.

Please fill out the receipt and wait for further instructions by the experimenter.
